# Self-induced optical non-reciprocity

**DOI:** 10.1038/s41377-024-01692-y

**Published:** 2025-01-02

**Authors:** Zhu-Bo Wang, Yan-Lei Zhang, Xin-Xin Hu, Guang-Jie Chen, Ming Li, Peng-Fei Yang, Xu-Bo Zou, Peng-Fei Zhang, Chun-Hua Dong, Gang Li, Tian-Cai Zhang, Guang-Can Guo, Chang-Ling Zou

**Affiliations:** 1 CAS Key Laboratory of Quantum Information & CAS Center For Excellence in Quantum Information and Quantum Physics, University of Science and Technology of China, Hefei, 230026 China; 2 State Key Laboratory of Quantum Optics and Quantum Optics Devices, and Institute of Opto-Electronics, Shanxi University, Taiyuan, 030006 China; 3 Collaborative Innovation Center of Extreme Optics, Shanxi University, Taiyuan, 030006 China

**Keywords:** Quantum optics, Photonic devices

## Abstract

Non-reciprocal optical components are indispensable in optical applications, and their realization without any magnetic field has attracted increasing research interest in photonics. Exciting experimental progress has been achieved by either introducing spatial-temporal modulation of the optical medium or combining Kerr-type optical nonlinearity with spatial asymmetry in photonic structures. However, extra driving fields are required for the first approach, while the isolation of noise and the transmission of the signal cannot be simultaneously achieved for the other approach. Here, we propose the mechanism of nonlinear non-reciprocal susceptibility for optical media and experimentally realize the self-induced isolation of optical signals without any external bias field. The self-induced isolation by the input signal is demonstrated with an extremely high isolation ratio of 63.4 dB, a bandwidth of 2.1 GHz for 60 dB isolation, and a low insertion loss of ~1 dB. Furthermore, the new mechanism allows novel functional optical devices, including polarization purification and non-reciprocal leverage. A complete passive isolator is realized by introducing an asymmetry cavity. It is demonstrated that the 70 *μ*W signal could lever the non-reciprocity and realize a 30 dB isolation of the backward laser with a power 100 times higher. The demonstrated nonlinear non-reciprocal medium provides a versatile tool to control light and deepen our understanding of light-matter interactions and enables applications ranging from topological photonics to unidirectional quantum information transfer in a network.

## Introduction

Optical non-reciprocity is of great importance due to the fundamental physics of light-matter interactions with broken time-reversal symmetry and their applications in non-reciprocal photonic devices^1–4^. For instance, non-reciprocity is highly desired in studies of topological photonic effects^5–7^ and could also be applied to unidirectional quantum information transfer in a network^8,9^. Two well-known routes are widely utilized to break the reciprocity of the optical system, i.e., the magneto-optical medium under an external magnetic bias and nonlinear optics effects^2,3,10^. However, regarding the practical difficulties accompanying magnetic fields and magneto-optic materials^11^, the nonmagnetic realization of non-reciprocity has aroused considerable research interest^12^ and has led to controversy^13,14^.

It has been experimentally demonstrated that reciprocity can be broken in photonic devices by combining Kerr-type optical nonlinearity of the signal with spatial asymmetry of the structure^15–18^. Although this approach allows passive asymmetric transmittance for forward and backward propagating light without any external bias, it is prevented from the realization of ideal optical isolation^19^ because the isolation of noise and the transmission of signal cannot be simultaneously achieved. Alternatively, ideal optical non-reciprocal phase shifting and isolation are possible by directional spatial-temporal modulation of the optical medium^1–3,20–23^. Utilizing nonlinear optical wave-mixing for coherent frequency conversion, a linear non-reciprocal susceptibility of the system with respect to the input signal can be realized by external drive fields. For instance, by either optical^24–29^, acoustic^30–33^ or microwave^34,35^ drives, directional conversion between the input signal and idle light can be realized. Since the underlying mechanism could be attributed to the phase-matching condition between traveling waves, this approach not only requires the drive fields and input signal in certain spatial modes but also imposes challenges in separating the signal from the drive field and idle outputs.

Here, we propose and demonstrate a new concept of nonlinear non-reciprocal (NLNR) susceptibility of the optical medium. Distinct from previous studies of magnetic-free non-reciprocity where nonlinear reciprocal effects are employed^19,26,36^, the new mechanism harnesses the intrinsic NLNR response, which allows ideal optical isolation with neither the requirements of an external bias field nor the phase-matching condition. The time-reversal symmetry of the NLNR medium is broken by the input signal itself, and the non-reciprocity property can be sustained and reconfigured by the transmitting signal, while the counter-propagating light is blocked simultaneously, where the self-induced isolation is a steady process that is essentially different from the self-induced transparency^37,38^. Remarkably, self-induced isolation with a 63.4 dB isolation ratio and a large bandwidth has been demonstrated. The self-induced non-reciprocity also brings novel functional optical devices, such as circular polarization purification and cavity-induced isolation through the non-reciprocal leverage effect. Our demonstration unveils new aspects of light-matter interactions and new physics in engineering optical non-reciprocal media, which could be extended to other atomic structures and spin systems in solids for functional acoustic and superconducting devices.

## Results

### Principle

**Fig. 1:**
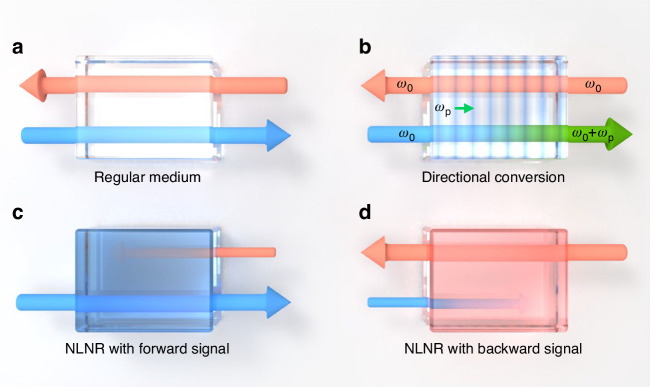
Schematic diagram of reciprocal and non-reciprocal optical media. **a** The regular medium that is transparent for both forward (blue arrow) and backward (red arrow) propagating light. **b** The medium under spatial-temporal modulation due to an external drive (*ω*_p_). The non-reciprocity is induced by the directional coherent conversion (*ω*_0_ → *ω*_0_ + *ω*_p_) for the forward signal. **c**, **d** Nonlinear non-reciprocal (NLNR) medium. The input signal induces non-reciprocal responses of the medium, so the direction of the isolation could be switched when changing the direction of the input signal

Figure 1 illustrates the reciprocal and non-reciprocal optical media. In Fig. 1a, the response of a regular medium to optical light, i.e., the linear susceptibility, is symmetric for forward and backward propagating beams. When utilizing the nonlinear optical susceptibility of the medium, an external directional driving field could induce the spatial-temporal modulation of the refractive index (Fig. 1b). Due to the phase-matching condition, the time-reversal symmetry is broken because the forward propagating signal is converted to idle frequency, while the backward propagating signal is free from nonlinear frequency conversion. In practice, the performance of this approach is limited by the imperfect conversion efficiency, and the bandwidth is limited by the strict phase-matching condition.

Figure 1c and d explain the proposed optical NLNR medium, whose response with respect to an input field (**E**) propagating along the *z*-direction (**e**_*z*_) can be written as^39^

1 $$\left(\begin{array}{c}{P}_{x}\\ {P}_{y}\end{array}\right)=\left(\begin{array}{cc}{\chi }_{xx}&i{\chi }_{xy}\\ -i{\chi }_{xy}&{\chi }_{yy}\end{array}\right)\left(\begin{array}{c}{E}_{x}\\ {E}_{y}\end{array}\right)$$

If *χ*_*xy*_ ≠ 0, the medium is non-reciprocal, as their optical susceptibility is anti-symmetric under time reversion, i.e., the refractive index for a *σ*^±^-polarized forward propagating light is different from that for a *σ*^∓^-polarized backward propagating light (see Supplementary Information for details). Such effects, which are also known as circular birefringence and circular dichroism magneto-optics effects^2^, govern the magnetic-based non-reciprocal components. In general, the non-reciprocal susceptibility could be expanded as

2 $${\chi }_{xy}={\chi }_{xy}^{(1)}{\bf{B}}\cdot {{\bf{e}}}_{z}+{\chi }_{xy}^{(3)}({\bf{E}}\times {{\bf{E}}}^{* })\cdot {{\bf{e}}}_{z}\,+\,\ldots$$

Here, the first term corresponds to the conventional linear magneto-optics effects due to an external bias magnetic field **B**. Other terms denote the nonlinear part of the susceptibility induced by the signal itself, and here, we focus on the second term that corresponds to the third-order NLNR susceptibility, which depends on the local spin property (**E** × E^*^) of the optical field^8^. It is worth noting that previous investigations of magnetic-free non-reciprocity are based on the nonlinear response of reciprocal components in optical medium’s susceptibility (Fig. 1b), while the NLNR is due to intrinsic nonlinearity of non-reciprocal susceptibility, which means that non-reciprocity based on NLNR medium does not need any external field.

Considering the circular dichroism (${\rm{Im}}({\chi }_{xy})$) of the NLNR material with thickness *L*, the corresponding power transmittance of the counter-propagating noise compared to that of the input is ${e}^{-4k{\rm{Im}}({\chi }_{xy})L}$ with the signal wave vector *k*, which leads to isolation. Comparing Fig. 1c, d, the isolation by NLNR is reconfigurable when changing the direction of the input. Similarly, the signal-induced circular birefringence ${\rm{Re}}({\chi }_{xy})$ would lead to a non-reciprocal phase $\phi =2k{\rm{Re}}({\chi }_{xy})L$, which enables the optical gyrator and circulator.

### Self-induced isolation

**Fig. 2:**
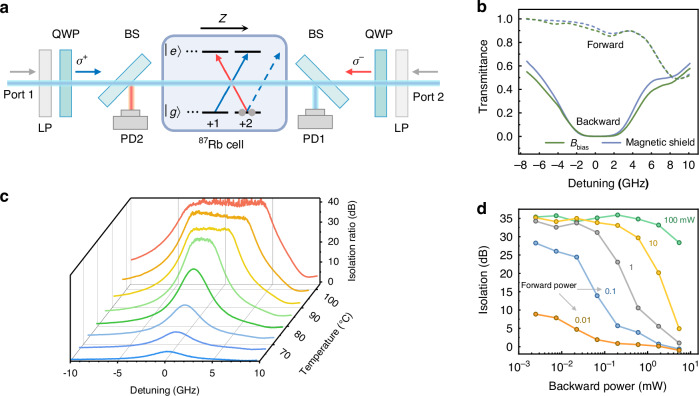
Experimental setup and characterization of the isolation capability. **a** Schematic of the experimental apparatus. LP linear polarizer, QWP quarter wave plate, BS beam spillter, PD photo detector. The kernel device of self-induced non-reciprocity is composed of a 10 mm Rb vapor cell filled with buffer gas, two LPs and two QWPs. The inset on the vapor cell denotes the energy structure of ^87^Rb, with the energy levels $\left\vert g\right\rangle$ and $\left\vert e\right\rangle$ denoted 5^2^*S*_1/2_ *F* = 2 and 5^2^*P*_1/2_ *F* = 2, respectively. Blue and red arrows represent the regulated *σ*^+^ and *σ*^−^ polarization of the forward and backward light, respectively. **b** Forward *σ*^+^ and backward *σ*^−^ transmission at 81 °C under two circumstances: applying a 5 Gauss bias magnetic field or using a magnetic shield. **c** Isolation spectra under different temperatures. The highest isolation 39 dB is reached when the temperature is >93 °C, and a 12.5 GHz bandwidth for 20 dB isolation is realized at 103 °C. For the results in both (**b**, **c**) the forward power is 100 mW, and the backward power is 10 *μ*W. **d** Maximum isolation ratio under different forward and backward powers at 84 °C. Colored numbers beside the lines represent the forward power: 0.01, 0.1, 1, 10, 100 mW

The NLNR is experimentally demonstrated with a ^87^Rb atom ensemble in a Rb vapor cell filled with nitrogen buffer gas (200 Torr). As schematically shown by the experimental apparatus in Fig. 2a, the two ports (1 and 2) are regulated by linear polarizers (LPs) and quarter-wave plates (QWPs). Due to the spin-dependent light-atom interaction at the microscopic level of each atom, the population of the atomic degenerated Zeeman ground sublevels could be polarized by the circularly polarized (*σ*^±^) input light, as shown by the energy diagram in Fig. 2a. The corresponding optical susceptibility of the atomic medium changes to be non-reciprocal and affects the transmittance of the light conversely. It is worth noting that the reconfiguration of an atomic medium could also be realized by other optical transitions coupling to the same ground sublevels, while additional external drive lasers are needed^40^. As shown in Fig. 2a, the forward input laser from Port 1 is *σ*^+^-polarized, and it reconfigures the NLNR medium by coupling to the D1 transitions of the ^87^Rb atoms (795 nm) without any external drives. Due to the selection-rule, the absorption of *σ*^+^-polarized input is inhibited when the atoms are polarized to the *m*_*F*_ = +2 state, while the *σ*^−^-polarized light could be attenuated when it passes through the cell. Consequently, the backward input at Port 2 with any polarization is blocked as it is either rejected from the LP or converted to *σ*^−^ and absorbed by the atoms.

**Fig. 3:**
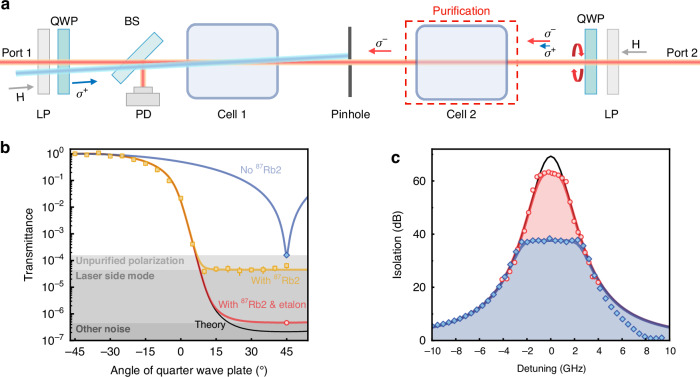
Ultrahigh isolation via optical circular-polarization purification. **a** Schematic of the improved experimental apparatus with an extra Rb vapor cell. The backward probe purified through Cell2 was used to characterize the isolation ratio in Cell1. **b** Transmittance of the backward probe against its polarization, which is controlled by the angle of the QWP (near port 2) with a forward signal power of 150 mW and a backward probe power of 1 mW. The zero angle corresponds to a linear polarization. The four lines show the corresponding theoretical predictions under different conditions, while the dots are the experimental results. Shaded areas denote noise floors from different causes. **c** The improved measurement of the isolation ratio (red circles) by using the NLNR effect for circular-polarization purification and an etalon to eliminate laser background noise, compared to the results without purification (blue diamonds). These two results correspond to *θ* = 45° in (**b**). The highest isolation ratio reaches 63.4 dB with a 2.1 GHz bandwidth for 60 dB isolation. The black line is the theoretical prediction of the ideal isolation ratio, while the red and blue lines are the results considering two different noise floors to fit the experimental data

**Fig. 4:**
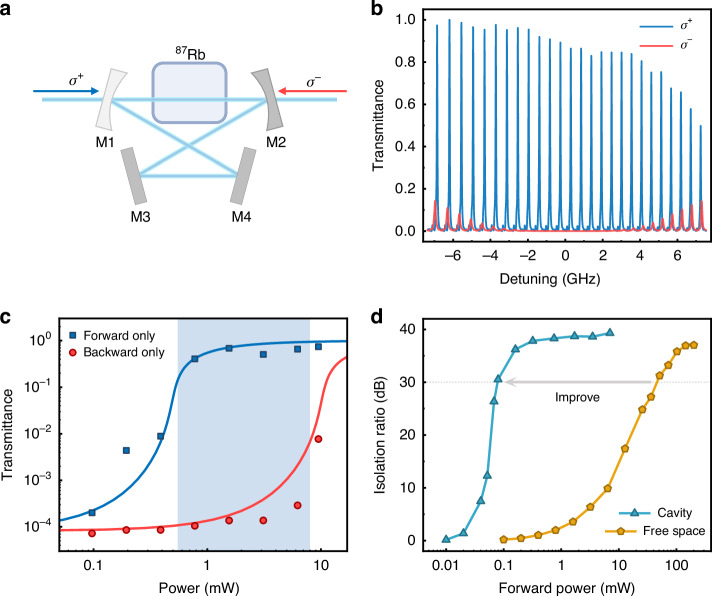
Cavity-induced non-reciprocal leverage. **a** The experimental apparatus of an asymmetric traveling wave cavity, comprising four mirrors and an Rb vapor cell inside. **b** Backward transmission spectra for the *σ*^+^ and *σ*^−^ probes when the cavity is resonantly driven by a forward signal laser. The powers of the forward laser and backward laser are both 7 mW. Here the effective cavity length is about *l* = 40 cm and the corresponding free spectral range (FSR) of the cavity is about 0.372 GHz. **c** Forward and backward transmission when only one laser is on. Solid lines are the theoretical prediction while dots correspond to the measured results. The blue shadow area indicates the power range where the cavity-induced isolation of the backward probe is effective even if there is no forward signal. **d** A contrast of the dependence of isolation on the forward signal power for cavity and free space configurations. The backward laser power is 7 mW for both lines and the data of free space configurations is measured separately, which has a certain deviation in temperature compared with Fig. 2d

Figure 2b demonstrates the non-reciprocal circular dichroism response of the medium by sending the signal transmitting forward through the system and simultaneously measuring the transmission of a backward probe from another laser with a similar frequency. The distinct contrast of the spectra for the forward and backward directions shows the excellent isolation of the backward probe over a broad frequency range, with the insertion loss of the forward input being only 0.5 dB. This small loss is unavoidable in hot atom ensembles due to that atoms are flying around and a certain forward laser power is consumed for polarizing the atoms that enter the laser beam area. We also noticed that the forward transmittance decreases when the laser is near-resonance with the transition between 5^2^*S*_1/2_ *F* = 1 and 5^2^*P*_1/2_ *F* = 2 at a positive detuning, which is attributed to non-saturable absorption of *σ*^+^-polarized light for this transition. Similar effect can also be observed in Fig. 3c and Fig. 4b. Here, to avoid the environment magnetic field-induced depolarization of the atom ground state populations, the cell is placed in a magnetic shield. Therefore, a practical isolator, normally used after a laser head, with forward and backward laser beam existing simultaneously is demonstrated.

For a concise investigation of the NLNR under different experimental conditions, we apply a very weak magnetic field *B*_bias_ ≈ 5 Gauss along the *z*-direction instead of the shield to simply the setup (Fig. 2a). Noting that weak *B*_bias_ is too weak to directly induce any observable non-reciprocity in our system, it could effectively protect the NLNR medium from stray magnetic fields, as shown by the spectra in Fig. 2b (see Supplementary Information for more details). The performance of the NLNR is characterized by ${\mathcal{I}}$, which is defined as the ratio of the backward transmission of *σ*^+^ and *σ*^−^. ${\mathcal{I}}$ is equivalent to the isolation ratio between forward and backward probes, and it holds the advantages in practice that the influence of the differences in optical insertion losses between forward and backward paths is mitigated.

Typical isolation ratios as a function of the probe laser frequency are shown in Fig. 2c. The density of the atoms is increased by ~2 orders of magnitude by varying the temperature of the cell from 61 °C to 103 °C^41,42^, and thus, the optical depth of the atomic ensemble is boosted. Both the isolation bandwidth for ${\mathcal{I}} \,>\, 20\,{\rm{dB}}$ and the maximum isolation ratio increase as the temperature increases. Thanks to the transition broadening due to the buffer gas in the vapor cell, an isolation bandwidth exceeding 12.5 GHz at 103 °C, (Doppler broadening <0.5 GHz at this temperature) which is orders of magnitude higher than the previous demonstration in atoms^25,27,40^. Another figure-of-merit for the isolation is the insertion loss, which is typically <0.5 dB below 90 °C and increases to 1.9 dB under 103 °C (see Supplementary Information for more details). In Fig. 2d, the dependence of ${\mathcal{I}}$ on the backward probe laser power *P*_*b*_ under different forward signal powers *P*_f_ is investigated. When the backward probe is strong enough, the NLNR effect due to the probe cannot be ignored, as the probe could cancel or overcome the non-reciprocal susceptibility induced by the signal. Therefore, a general trend of the degradation of ${\mathcal{I}}$ is observed for larger *P*_*b*_/*P*_*f*_.

### Non-reciprocal purification and leverage

The flat-top spectra for the dense NLNR medium shown in Fig. 2c indicate a much higher ${\mathcal{I}}$ around zero detuning hindered by noise, which doesn’t change with the susceptibility. When the *σ*^−^-polarized signal is almost completely absorbed by the NLNR medium, the remaining *σ*^+^-polarized light and stray light leads to the flat-top spectra (see Supplementary Information). After a thorough inspection of the system, we found that the measured ${\mathcal{I}}$ is essentially limited by the imperfect polarizer and waveplate (Fig. 2a), which generates an impure circular polarization, i.e., the backward probe contains the *σ*^+^ component of a portion around 10^−4^ (100 ppm). As shown by the setup in Fig. 3a, we use an additional NLNR medium to purify the polarization of the probe. Specifically, a strong enough (1 mW) backward *σ*^−^ probe, with a small portion of the *σ*^+^ component in it, will polarize the medium in Cell2 to only absorb *σ*^+^ light and thus purifies itself. Figure. 3b presents the transmittance of the probe for different input polarizations controlled by the angle of the QWP. Treating the self-induced isolation (left half of the setup) as a black box that only *σ*^+^-polarized light can pass through, we can expect a transmittance of ${\cos }^{2}(\theta +\frac{\pi }{4})$ without Cell2. However, the NLNR effect in Cell2 gives a significantly different response, as the measured transmittance of the laser is 0.02 when *θ* = 0, in contrast to 0.5 from ${\cos }^{2}(\frac{\pi }{4})$. The observed purification is in excellent agreement with theoretical fitting (see Supplementary Information) and reveals other imperfections due to laser background noise. By significantly improving the circular polarization purity and filtering the background noises, we achieved an ultrahigh isolation ratio of 63.4 dB, with a 60 dB isolation bandwidth as large as 2.1 GHz in Fig. 3c. The asymmetry lineshape is attributed to the presence of transitions associate with *F* = 2 ground states, which enhances absorption on the blue-detuned side of the target transitions. The detected flat-top spectra imply that our NLNR provides an ultrasensitive circular-polarization analyzer, where the ratio of imperfect component can be obtained by the measured isolation and ideal isolation (see Supplementary Materials for more details), and it also suggests a potential application of the NLNR device for optical circular-polarization purification. The experimental spectra are consistent with our theoretical model, which predicts a purity of the circular polarization better than 0.5 ppm, the achievable isolation is 70 dB.

It is anticipated that the direct application of the NLNR might be limited to scenarios where the input signal power is strong enough and the backward light is much weaker. Harnessing the inherent nonlinearity of the effect, this drawback could be mitigated by non-reciprocal leverage. As shown in Fig. 4a, by placing the NLNR medium into an asymmetric cavity with mirrors of different reflectivities, the optical isolation could be leveraged from two aspects. First, the intracavity NLNR could be enhanced by the resonance, thus the forward signal power to activate the medium to be transparent is lowered. Second, the asymmetric cavity containing the Kerr-like ${\chi }_{xy}^{(3)}$ nonlinear medium allows dynamic non-reciprocity^15–17^. Figure 4b presents the backward transmission spectra for the *σ*^+^ and *σ*^−^ probes when the cavity is resonantly driven by a forward signal laser. The mirror transmittances are 0.082 (M1) and 0.004 (M2), respectively, and the laser power is 7 mW for both cases. The shifts between the forward and backward resonances at large detunings are due to the circular birefringence effect, which is from the effective dispersion coupling between the cavity and the atom^40^.

The power-dependence relation of the resonance transmittance around zero detuning for both directions is tested in Fig. 4c. It is found that leverage could be activated when *P*_F_ > 400 *μ*W, but the backward light is blocked as long as its power *P*_B_ < 8 mW. The non-reciprocal leverage effect is manifested by testing the isolation ${\mathcal{I}}$ when the non-reciprocal leverage is sustained by an input *P*_F_, with a fixed backward *P*_B_ = 7 mW. We found that ${\mathcal{I}}$ could be as large as 30 dB even when *P*_F_ = 0.01*P*_B_, as shown in Fig. 4d. Compared with the NLNR medium in free space, the leverage shows a 3 orders of magnitude improvement on the requirement of *P*_F_ for ${\mathcal{I}}=30\,{\rm{dB}}$. It is important to note that the configuration of the heating belt and temperature sensor were slightly modified between the independent measurements presented in Figs. 2 and 4. As a result, there are minor deviations in the actual cell temperatures for the data shown in the different figures. Consequently, slight discrepancies in the isolation values can be observed when comparing the results in Fig. 2d and Fig. 4d.

## Discussion

By exploiting the nonlinear non-reciprocal susceptibility of the optical medium, the non-reciprocity could be realized by the input signal itself and leveraged by spatial asymmetry, without requirements for any external driving field or a strict phase-matching condition for the input. Benefiting from the combined nonlinearity and non-reciprocity, novel photonic devices, such as circular polarization filters, are enabled. In particular, two schemes of optical isolations based on the NLNR, for the atomic ensemble medium in free space and in cavity, are proposed and experimentally investigated. We note that both the free-space and cavity schemes studied in this work are significantly different from previous demonstrations. In Table 1, we summarized different mechanisms for realizing optical isolation and compared them from the aspects of the requirement on external bias field and the dependence on the input signal. The mechanisms based on Faraday effect^11^, coherent frequency conversion^1–3,9,20–35^, and the optically-induced magnetization^40^ are very similar to each other, and these schemes requires external bias field, while the dynamics non-reciprocity^15–19^ could work without bias field if there is no signal input. The self-induced isolation in free-space is distinct from all previous demonstrations, but it is limited to working under the condition that backward stray light is much weaker than the input signal. Beneficial from the non-reciprocal leverage effect, our scheme with a cavity could overcome the drawback of self-induced non-reciprocity in free-space, and achieves a complete passive isolation. As shown by the blue shadow area in Fig. 4c, the backward signal can be isolated when there is no forward signal.

**Table 1:** Comparison among different isolation mechanisms about the working conditions: no external bias field, with forward signals passing through and without forward signals

Mechanisms	Max isolation ratio (dB)	insertion loss (dB)	no external field	w/ signals	w/o signals
Faraday effect	>60	<1	✗	*✓*	*✓*
coherent conversion^46^	38	19	✗	*✓*	*✓*
OIM^40^	52	~20	✗	*✓*	*✓*
Kerr effect^10^	24	7	*✓*	✗	*✓*
self-induced isolation	63	1	*✓*	*✓*	✗
leveraged isolation	39	~20	*✓*	*✓*	*✓*

*OIM* optically-induced magnetization. Self-induced isolation and leveraged isolation correspond to the free-space and the cavity schemes in this work, respectively

Our experiments accomplish a new route for realizing functional and high-performance non-reciprocal photonic devices. Notably, the principle of NLNR validated in rubidium gas can be easily extended to other atoms and molecules for non-reciprocity at UV, mid-infrared or THz frequencies and thus could also be implemented by integrated photonic structures, such as atom-cladded waveguide^43^ and nanofiber^44^ or solid state emitter-doped photonic structures^45^, and so on. For example, the transitions between 5^2^*S*_1/2_ and 6^2^*P*_1/2_ in Rb (422 nm), between 6^2^*S*_1/2_ and 6^2^*P*_1/2_ in Cs (895 nm) and between 6^2^*S*_1/2_ and 7^2^*P*_1/2_ in Cs (459 nm) are also suitable for realizing self-induced isolation. Furthermore, any medium with transitions from a lower energy level with angular momentum *L* to an upper energy level with angular momentum *L* − 1 or *L* can be used to achieve self-induced non-reciprocity. In the future, our setup could be minimized to realize compact, passive, and high-performance devices, which could replace commercial products when using semiconductor lasers in the studies of atomic and molecular physics. Furthermore, the inherent non-reciprocal nonlinear susceptibility could be generalized to other nonlinear effects, such as the cross-Kerr and coherent frequency mixing processes. Therefore, this work presents a significant conceptual advance in optics and could stimulate further exploration of physics with nonlinear dynamics and non-reciprocity by considering the interplay between the optical fields, microwave fields, and configuration (internal state population) of the medium.

## Materials and methods

### Experimental setup

The detailed experimental setup for the self-induced optical non-reciprocity for the cavity-free scenario is illustrated in Supplementary Fig. S1. For the study of cavity-induced leverage, a bow-tie cavity is added to the system by inserting four mirrors around the Rubidium vapor cell, with the bow-tie cavity sketched in Fig. 4a in the main text. The reflectivities of the four mirrors are *R*_1_ = 91.8% (M1), *R*_2_ = 99.6% (M2), and *R*_3_ = *R*_4_ ≈ 100%(M3, M4). In the setup, there are two lasers: Laser1 (Toptica DLpro 795 nm) provides the backward probe, which passes through the vapor cell with a beam waist of 600 *μ*m, and Laser2 (Wavicle ECDL 795 nm) provides the forward signal with a beam waist of 750 *μ*m. Two quarter wave plates have two mutually perpendicular fast axes, thus the forward and backward light have orthogonal circular polarization when interacting with the atoms in the vapor cell. Meanwhile, the forward (backward) horizontally polarized light can travel from port1(2) to port2(1) without reflection on any polarization beam splitter. Therefore, the setup allows the measurement of the non-reciprocity properties of our devices. More details about the characterization of the non-reciprocity are provided in Supplementary Materials.

In our experiments, the frequencies of the two lasers are tuned independently and are near resonance with the D1 transitions of the rubidium atoms (detuning <10 GHz). For the measurement of the forward and backward transmittance of light, there is potential cross-talk between the lights that induces difficulties in measuring ultrahigh isolation ratios due to the backgrounds. Therefore, the lock-in amplifier technique is employed in these measurements. We use a chopper (Thorlabs MC2000) and a lock-in instrument (Zurich MFLI 500 kHz) to suppress the background noise and electrical noise on the optical detectors (Thorlabs PDA36A2 & APD410A).

### Experimental devices

The atomic vapor cell we used is a 10 mm cube filled with ^87^Rb atoms and 0.23 amg (200 Torr at room temperature) N_2_ as the buffer gas. The buffer gas is beneficial for our device from two aspects. First, it changes the motion of ^87^Rb atoms from linear motion to Brownian motion, thus the depolarization of the ^87^Rb atoms is suppressed. Additionally, the transient effect is suppressed, and the atoms can be polarized more efficiently. Second, the collision between excited ^87^Rb atoms and N_2_ molecules will greatly increase the absorption linewidth, which will improve the isolation bandwidth (~10 GHz) when compared to the case without buffer gas. The isolator is robust to the conditions of the vapor cell, including the cell length, the pressure of the buffer gas as well as the purity of the atom gas. Slight differences in the conditions of the vapor cell will cause variations in the isolator’s performance, but it will still function as a high-performance isolator.

Since an external magnetic field would induce the depolarization of the ground states of the atoms, the performance of the self-induced non-reciprocity degrades by the background magnetic fields. Therefore, two different approaches are applied in our experiments to mitigate the influence of the background magnetic field: (1) place the atomic vapor cell inside a magnetic shield, which is a Permalloy cylinder (thickness of 0.5 mm), or (2) apply a very weak bias field along the propagation direction of the signal. By the first approach, the magnetic-free nature of the self-induced non-reciprocity mechanism is experimentally demonstrated, as shown by Fig. 2b in the main text. We found a slight difference between the transmission under the magnetic shield and bias field through Fig. 2b. The transmission rates of forward *σ*^+^ signals are almost the same under different temperatures, which provides additional evidence that the only effect of *B*_bias_ = 5 G is to eliminate stray magnetic fields in the environment, not to induce non-reciprocity. Since the magnetic shield brings difficulties when changing the working temperature of the vapor cell and when working at different optical configurations, we carry out further systematic experimental characterizations of the device under different conditions with a bias magnetic field for convenience.

### Characterization of the isolation ratio

The isolation ratio is an important quantity for practical applications of non-reciprocal devices. Usually, the isolation ratio (${\mathcal{I}}$) is defined as the ratio between the forward transmittance and the backward transmittance of light. When measuring the transmittance of light, we could place BS into the optical paths to measure the light intensity. The BSs are placed just close to the cell because the atomic medium provides the essential ingredients for realizing non-reciprocity and because the polarization of forward and backward light are orthogonal and the possible reflections of input signal and noises introduced by other optical components could be reduced. Therefore, close to the vapor cell, the transmittance of forward *σ*^+^-polarized light (*T*_f,+_) and the transmittance of backward *σ*^−^-polarized light (*T*_b,-_) could be measured separately by two PDs, and the system isolation ratio could be derived as

3 $${{\mathcal{I}}}_{{\rm{sys}}}=10\times {{\rm{Log}}}_{10}\left(\frac{{T}_{{\rm{f}},+}}{{T}_{{\rm{b}},-}}\right)$$

However, when characterizing the transmittance of light in real experiments, the optical paths and the optical components (including the BSs and detectors) are different for forward and backward light. Therefore, the different optical paths and components could introduce different losses to the transmittance, which is difficult for calibrations of transmittance and eventually causes errors in the estimated ${\mathcal{I}}$.

For a more precise characterization of the ${\mathcal{I}}$ and to avoid the difficulties in calibrations, we characterize the ${\mathcal{I}}$ by measuring the ratio between the transmittance for backward propagating probe light with different polarizations (*σ*^+^- or *σ*^−^-polarized light when passing through the cell). We adapt the property of the atomic medium that the circular dichroism or birefringence property of the vapor cell is the same for both forward and backward light, i.e., the transmittance of the *σ*^+^-polarized light through the cell should be exactly the same for both the forward and backward directions. Therefore, *T*_f,+_ can be obtained by equivalently measuring *T*_b,+_. The optical configuration for measuring *T*_b,-_ and *T*_b,+_ are exactly the same except that the angle of the QWP is rotated by 90°. Therefore, the potential calibration errors are significantly suppressed when measuring transmittance light by sharing the same optical path and photodetector (see Supplementary Materials for more details), and the corresponding isolation ratio is

4 $${\mathcal{I}}=10\times {{\rm{Log}}}_{10}\left(\frac{{T}_{{\rm{b}},+}}{{T}_{{\rm{b}},-}}\right)\approx {{\mathcal{I}}}_{{\rm{sys}}}$$

## Supplementary information

Supplementary Information for “Self-induced optical non-reciprocity”

## Data Availability

All data generated or analysed during this study are available within the paper and its Supplementary Information. Further source data will be made available on reasonable request.

## Code availability

The code used to solve the equations presented in the Supplementary Information will be made available on reasonable request.
