# Supplementary material for: Self-induced optical non-reciprocity
Source: Light Sci Appl. 2025 Jan 2;14:23. doi: 10.1038/s41377-024-01692-y (PMC11693750; doi:10.1038/s41377-024-01692-y)
Supplement: Supplementary file 1 — Supplementary Information for “Self-induced optical non-reciprocity” [file 41377_2024_1692_MOESM1_ESM.pdf]

Supplementary Information for "Self-induced optical non-reciprocity"

Zhu-Bo Wang,[1,*] Yan-Lei Zhang,[1,*] Xin-Xin Hu,[1] Guang-Jie Chen,[1] Ming Li,[1]
Peng-Fei Yang,[2,3] Xu-Bo Zou,[1] Peng-Fei Zhang,[2,3,†] Chun-Hua Dong,[1,‡]
Gang Li,[2,3,§] Tian-Cai Zhang,[2,3] Guang-Can Guo,[1] and Chang-Ling Zou[1,2,¶]

[1] *CAS Key Laboratory of Quantum Information & CAS Center For Excellence in Quantum Information and Quantum Physics, University of Science and Technology of China, Hefei 230026, China.*
[2] *State Key Laboratory of Quantum Optics and Quantum Optics Devices, and Institute of Opto-Electronics, Shanxi University, Taiyuan 030006, China*
[3] *Collaborative Innovation Center of Extreme Optics, Shanxi University, Taiyuan 030006, China.*

---

* These two authors contributed equally to this work.
† zhangpengfei@sxu.edu.cn
‡ chunhua@ustc.edu.cn
§ gangli@sxu.edu.cn
¶ clzou321@ustc.edu.cn

CONTENTS

I. Experimental details S3
A. Experimental setup for free-space measurement S3
B. Characterization of the isolation ratio S3
C. More results about the performance of self-induced non-reciprocity S4
D. Circular polarization purification S5
E. Parameters of the cavity S6

II. Theoretical derivations S8
A. The microscopic theory S8
B. The macroscopic description S9
C. Non-reciprocal light propagation S11
D. Cavity-enhanced non-reciprocity S13
E. Design of the optical circulator based on the NLNR medium S13
F. Numerical Results S15
1. Self-induced non-reciprocity without cavity S15
2. Cavity-enhanced non-reciprocal leverage S16

References S18

I. EXPERIMENTAL DETAILS

A. Experimental setup for free-space measurement

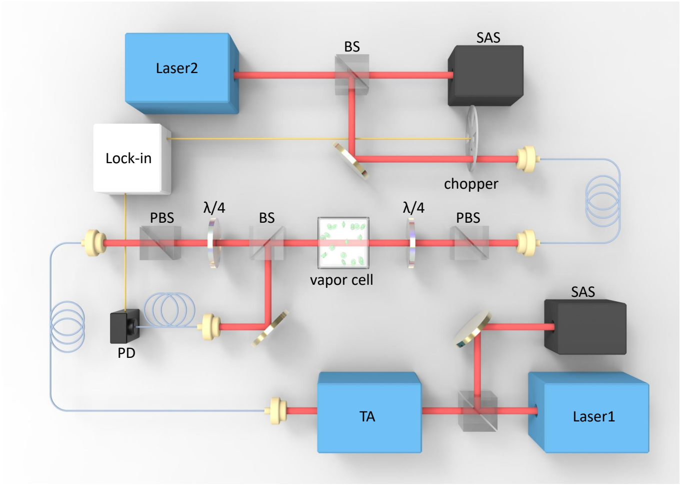

Figure S1. **Schematic of the experimental setup for the isolation measurement without a cavity.** The red beams denote the free-space optical paths of both the forward signal and backward probe, the gray lines represent the optical fibers, and the yellow lines are electric cables. SAS: saturated absorption spectrum. TA: tapered amplifier. PBS: polarization beam splitter. BS: beam splitter. QWP: quarter wave plate.

B. Characterization of the isolation ratio

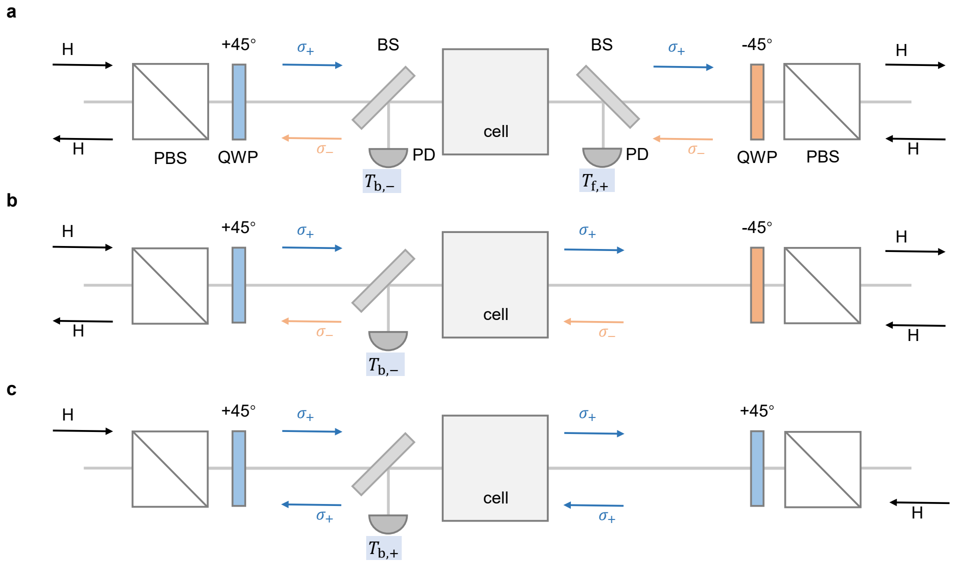

Figure S2. **Definition of the isolation ratio. a**, The system isolation ratio is defined as the ratio between the forward transmittance and the backward transmittance. $T_{\mathrm{f(b)},\pm}$ is the transmission of the forward (backward) laser with an H-polarization from the input and output ports. The polarization of the light when coupled with the atoms depends on the incident direction due to the quarter wave plates. **b**,**c**, The experimentally measured isolation ratio is defined as the transmittance ratio between the backward and forward probes that are σ^+-polarized and σ^--polarized when coupled with the atoms.

C. More results about the performance of self-induced non-reciprocity

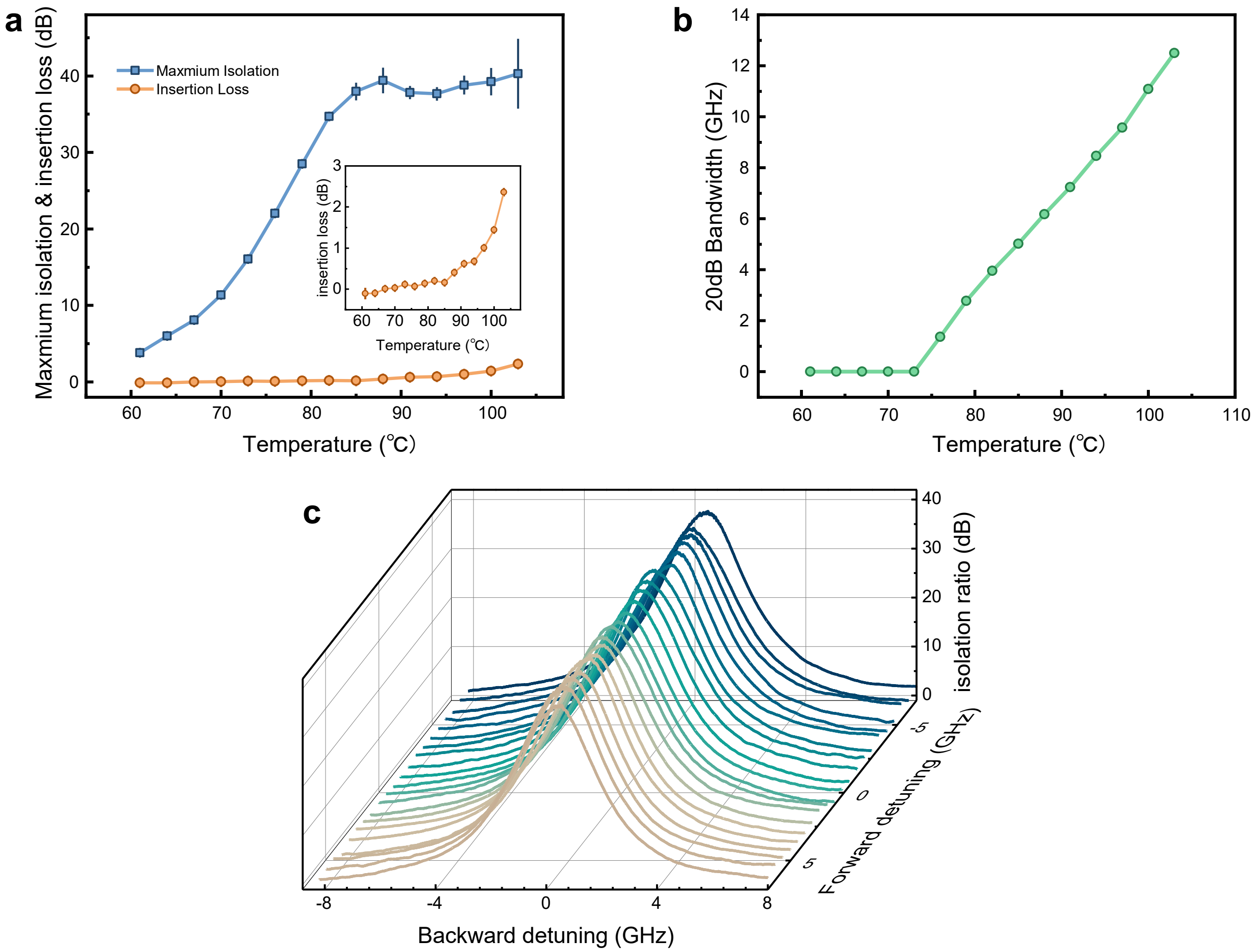

Figure S3. Detailed information of the isolator. **a**, Maximum isolation ratio (blue) and insertion loss (orange) for different cell temperatures. **b**, 20 dB isolation bandwidth for the backward probe under different temperatures with the forward laser locked on resonance. **c**, Isolation spectra under different forward laser detunings. All the spectra are measured under 82 °C, with a 100 mW forward laser and a 10 μW backward laser.

The temperature dependence of the isolation ratio of the device is investigated, and the results are summarized in Figs. S3a and b. The maximum isolation ratio $\mathcal{I}_{\max}$ increases with the temperature from 61 °C to 85 °C, and the $\mathcal{I}_{\max}$ saturates at higher temperatures because the signal is immersed into the background noises, as discussed in the main text (Fig. 3b and c). As shown by the yellow dots, the insertion loss of the device (the loss of forward signal) also increases with temperature, which could be attributed to the higher density of the atomic medium and thus larger absorption. At a reasonable working temperature below 97 °C, the insertion loss is lower than 1 dB. In addition, the 20 dB isolation bandwidth also increases with temperature except for 61 $\sim$ 73 °C, where the optical density of the atom vapor is too low and the corresponding $\mathcal{I}_{\max}$ is lower than 20 dB.

All the transmission spectra in the main text are measured with the forward laser being resonant to the transition between $5^2S_{1/2}$ $F = 2$ and $5^2P_{1/2}$ $F = 2$. For potential applications in practice, the device might work for input signal and backward noises with different frequencies. Therefore, we also characterized the device by measuring its isolation at different forward signal detunings and backward probe detunings. As shown in Fig. S3c, different colored lines denote the backward isolation spectrum (isolation ratio against the backward probe detuning) under different forward laser detuning. For the input signal detuning varying from -7 GHz to 7 GHz, it is difficult to tell the difference between these spectra. We notice that the corresponding working frequency range for the forward signal is as large as

$\sim 14\,\mathrm{GHz}$, which is much higher than the bandwidth (less than $4\,\mathrm{GHz}$) of the $20\,\mathrm{dB}$ isolation ratio for the backward probe. Such a high performance of the forward signal detuning could be attributed to the nonlinear nature of the self-induced non-reciprcity, i.e., the atom ensemble can be effectively polarized by the strong forward signal even when the detuning is as large as $7\,\mathrm{GHz}$.

D. Circular polarization purification

The apparatus for the study of polarization purification by the self-induced non-reciprocity mechanism in an extra atomic vapor cell (Cell2, a new cell with a length of 75 mm filled with 50 Torr nitrogen buffer gas) is shown in Fig. 3a of the main text. In our experiments, the backward probe (1 mw) and the forward signal (150 mw) are only overlapped in Cell1 for the studies of non-reciprocity, and the two laser beams have a small angle ($\sim 3^\circ$) to guarantee that the forward signal can be blocked and cannot reach Cell2 to avoid its influence on the polarization purification process. Cell1 (same one used in Fig. 2 in the main text) is heated to $84\,^\circ\mathrm{C}$, while the temperature of Cell2 is $65\,^\circ\mathrm{C}$.

In this configuration, Cell1 serves as the optical isolator that only the σ^+-polarized light can pass through, with a transmittance for σ^+-polarized light of $T_+^{(1)} \approx 1$, as the insertion loss is negligible (Extended Data Fig. 2a). In contrast, the transmittance for σ^--polarized light is approximately $T_-^{(1)} \approx 10^{-\mathcal{I}_{\mathrm{ideal}}/10}$, which is determined by the ideal isolation ratio $\mathcal{I}_{\mathrm{ideal}}$ induced by the atomic medium. However, the ideal isolation ratio $\mathcal{I}_{\mathrm{ideal}}$ could not be directly measured experimentally due to the imperfect polarization control of the probe laser. For an input laser with an intensity of I and the polarization controlled by the QWP angle θ, the intensity of the $\sigma^\pm$ polarization is

$$I_\pm = I\cos^2\left(\theta \pm \frac{\pi}{4}\right). \tag{S.1}$$

Assuming the transmittance of Cell2 for $\sigma^\pm$ polarization is $T_\pm^{(2)}(I_+, I_-)$, which is a function of the input laser intensities, the transmittance of the backward probe can be derived as

$$T = \frac{T_+^{(1)} \times I_+ T_+^{(2)}(I_+, I_-) + T_-^{(1)} \times I_- T_-^{(2)}(I_+, I_-)}{I_+ + I_-} \tag{S.2}$$

$$\approx \frac{I_+ T_+^{(2)}(I_+, I_-) + I_- 10^{-\mathcal{I}_{\mathrm{ideal}}/10} T_-^{(2)}(I_+, I_-)}{I} \tag{S.3}$$

$$= \cos^2\left(\theta + \frac{\pi}{4}\right) T_+^{(2)}(I_+, I_-) + \sin^2\left(\theta + \frac{\pi}{4}\right) 10^{-\mathcal{I}_{\mathrm{ideal}}/10} T_-^{(2)}(I_+, I_-). \tag{S.4}$$

When Cell2 is in absence, for the ideal case that $T_\pm^{(2)}(I_+, I_-) = 1$, the expected transmitted backward probe light

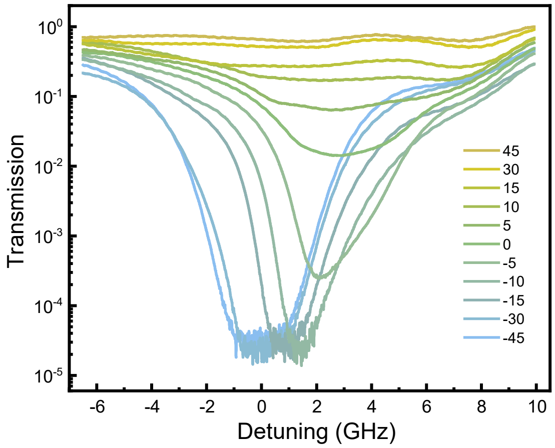

Figure S4. Transmission spectra of the backward laser after purification and isolation for different angles of the QWP. Angle $+45^\circ$ means the backward laser has a polarization of σ^+, while angle -45° corresponds to σ^-.

is

$$T_{\rm ideal} = \cos^2\left(\theta+\frac{\pi}{4}\right)+\sin^2\left(\theta+\frac{\pi}{4}\right)10^{-I/10}. \tag{S.5}$$

By tuning θ, the ideal isolation ratio can be solved by comparing the minimum and maximum transmittance of the backward probe light as $\mathcal{I} = -10\mathrm{Log}_{10}\left[10^{-\mathcal{I}_{\rm ideal}/10}\right] = \mathcal{I}_{\rm ideal}$.

From the experimental results of the θ-dependence of the transmittance, as shown in Fig. 3b of the main text, excellent agreement with the prediction is observed. However, the minimum transmittance is limited by some background noise and limited precision of θ tuning. By introducing the achievable $\theta+\frac{\pi}{4}=\delta\theta$ approach 0 and the background noise $I_{\rm bg}$, the measured maximum isolation ratio can be solved experimentally as

$$\mathcal{I} = -10\mathrm{Log}_{10}\left[I_{\rm bg}/I+\cos^2(\delta\theta)+10^{-\mathcal{I}_{\rm ideal}/10}\right]. \tag{S.6}$$

Therefore, the measured $\mathcal{I}$ is lower than the expected isolation for the ideal case. For a characterization of the ideal isolation ratio achievable by our self-induced non-reciprocity mechanism, we introduced the lock-in technique and spectrum filtration of the light to suppress the background noise; however, the measured I still deviated from the ideal value, as indicated by the flat-top isolation spectrum measured in our experiments. Therefore, the main obstacle for achieving $\mathcal{I} > 40\,\mathrm{dB}$ is mainly attributed to the imperfect circular polarization of the probe light.

If we ignore the background noise, the device can be used as a circular polarization analyzer. Here we assume that the initial polarization is $I = I_+ + I_-$ and I_- is absorbed after passing the cell, and the corresponding measured isolation can be written as

$$\mathcal{I}_{\rm measured} = -10\mathrm{Log}_{10}\frac{I_+ + I_- 10^{-\mathcal{I}_{\rm ideal}/10}}{I_+ + I_-}. \tag{S.7}$$

It is obvious that we have $\mathcal{I}_{\rm measured} = \mathcal{I}_{\rm ideal}$ when $I_+ = 0$. If the polarization is not pure, we can obtain the ratio

$$I_+/I_- = \left(10^{-\mathcal{I}_{\rm measured}/10} - 10^{-\mathcal{I}_{\rm ideal}/10}\right)/\left(1-10^{-\mathcal{I}_{\rm measured}/10}\right), \tag{S.8}$$

where the $\mathcal{I}_{\rm ideal}$ can be obtained by the theoretical fitting or the experimental calibration. In general, we can obtain the impurity $I_+/I_- \approx 10^{-\mathcal{I}_{\rm measured}/10}$ when $\mathcal{I}_{\rm ideal} \gg \mathcal{I}_{\rm measured} \gg 10$ for the practical experiment. For the flat-top spectra, it is also attributed to the polarization impurity, where the corresponding transmission can be written as $T = \left(I_+ + I_- 10^{-\mathcal{I}_{\rm ideal}/10}\right)/\left(I_+ + I_-\right)$. We can obtain a constant value $T \approx I_+/\left(I_+ + I_-\right)$ when $I_- 10^{-\mathcal{I}_{\rm ideal}/10} \ll I_+$, which leads that the flat-top spectra.

By introducing the extra vapor cell for the probe laser, the self-induced non-reciprocity mechanism could induce a nonlinear response of the $T_\pm$ that depends on the power contrast between the two circular polarizations for a given total intensity of the backward laser of 1 mw. The backward probe laser is strong enough to polarize the atom in Cell2, while it is too weak to change the population of the atoms in vapor cell1 due to a much stronger signal laser (150 mW). Therefore, we can realize $T_-^{(2)} \approx 1$ and $T_+^{(2)} \ll 1$ or reversely $T_+^{(2)} \approx 1$ and $T_-^{(2)} \ll 1$. Therefore, when the angle of the QWP is close to $\theta = -\pi/4$ (i.e., $\delta\theta \approx 0$), we have $T \approx I_{\rm bg}/I + 10^{-\mathcal{I}_{\rm ideal}/10}$. Thus, the measured isolation ratio is essentially limited by the background noise, and the influence due to imperfect circular polarization control is mitigated.

Figure S4 presents the detailed spectra of the backward probe laser after it is transmitted through both Cell2 and Cell1 sequentially. The maximum isolation is extracted from these spectra and presented in Fig. 3b of the main text. In Fig. S4, there is a resonance frequency shift between -45° and 0°. From Eq. (S.4), for the QWP angle of 0°, the input to Cell1 is linearly polarized, and the detected spectrum is contributed by $T_+^{(2)}$. For the QWP angle of -45°, the detected spectrum is mainly contributed by Cell1. Therefore, the frequency shift should be explained by the different absorption centers of the two cells, which is caused by different pressures of the buffer gas in Cell1 and Cell2 [1].

E. Parameters of the cavity

The non-reciprocal optical leverage could be realized by placing Cell1 into an asymmetric cavity. The setup is illustrated in Fig. 4a in the main text. The forward and backward ports of the cavity are flat mirrors, with reflectivities of $R_1 = 91.8\%$ and $R_2 = 99.6\%$. According to the relation $e^{-\kappa_{1(2)}L_{\rm rt}/c} = \sqrt{R_{1(2)}}$, where $L_{\rm rt}$ is the cavity round trip length and c is the vacuum light velocity, the corresponding external coupling strengths of the cavity ports

are $\kappa_1/2\pi = 3.1\,\mathrm{MHz}$ and $\kappa_2/2\pi = 0.15\,\mathrm{MHz}$, respectively. According to the two-port cavity input-output relation, we obtain the transmittance of the cavity as

$$T_{\mathrm{cav},\pm} = \left| \frac{2\sqrt{\kappa_1\kappa_2}}{-i(\omega_{\mathrm{c},\pm} - \omega_p) - \kappa_1 - \kappa_2 - \kappa_0 - \kappa_\pm} \right|^2, \tag{S.9}$$

where the subscript $\pm$ denotes the circular polarization, κ_0 is the intrinsic cavity dissipation rate due to the mirror surface roughness and the absorption and the scattering due to the cell, and $\kappa_\pm = \ln(T_\pm)c/2L_{\mathrm{rt}}$ is the corresponding atomic absorption loss rate due to the vapor cell for $\sigma^\pm$-polarized light. Note that due to the potential circular birefringence and dichroism, the atomic media might induce polarization-dependent absorption loss $\kappa_\pm$ and cavity resonance frequency $\omega_{\mathrm{c},\pm}$. For forward and backward optical powers of $P_{\mathrm{in,f(b)}}$, the corresponding transmitted power of the cavity is $P_{\mathrm{in,f(b)}}T_{\mathrm{cav},+(-)}$, which should equal the output of the cavity field as $(1-R_{2(1)})P_{cav,f(b)}$, and the intracavity light intensities are

$$P_{\mathrm{cav,f}} = \frac{1}{1-R_2}P_{\mathrm{in,f}}T_{\mathrm{cav},+}, \tag{S.10}$$

$$P_{\mathrm{cav,b}} = \frac{1}{1-R_1}P_{\mathrm{in,b}}T_{\mathrm{cav},-}. \tag{S.11}$$

Comparing the expressions of the intracavity laser power for the forward and backward input, we find that (1) if the cavity is symmetric $R_1 = R_2$, the intracavity power could be significantly different when $\kappa_+ \neq \kappa_-$, which is induced by the circular dichroism of the atomic medium under a circularly polarized driven field. (2) The difference between the intracavity power could be further enhanced by the asymmetric cavity $R_1 \neq R_2$.

We should note that the above derivations of the cavity field and transmittance only valid for the case that the total cavity dissipation rate $\kappa_1 + \kappa_2 + \kappa_0 + \kappa_\pm$ is small when compared with the free-spectral range of the cavity. For example, when the $\kappa_+ \approx 0$ as the atomic medium is transparent ($T_+ \approx 1$) for strong forward interactivity σ^+-polarized signal power, then we should have the on-resonance transmittance of the forward signal by the above expression as

$$T_{\mathrm{cav},+} = \frac{1}{1-R_2}\frac{4\kappa_1\kappa_2}{(\kappa_1+\kappa_2+\kappa_0)^2}. \tag{S.12}$$

and the corresponding intracavity power is

$$P_{\mathrm{cav},+} = P_{\mathrm{in,f}}\frac{1}{1-R_2}\frac{4\kappa_1\kappa_2}{(\kappa_1+\kappa_2+\kappa_0)^2}. \tag{S.13}$$

In contrast, the transmittance for the backward probe is

$$T_{\mathrm{cav},-} = (1-R_1)(1-R_2)\times 10^{-\mathcal{I}_{\mathrm{ideal}}/10}, \tag{S.14}$$

which should be approximately 4 orders of magnitude weaker than the case of free space, and the intracavity laser power that enters the cell should be

$$P_{\mathrm{cav},-} = (1-R_2)P_{\mathrm{in,b}}. \tag{S.15}$$

Comparing the expressions for the cavity-enhanced and free-space cases, we find that the cavity has the following advantages:

1. The isolation ratio is enhanced as the non-reciprocal light-atom interaction is enhanced by the resonances.

2. The asymmetric cavity enables distinct intracavity power for a leverage effect. For a relatively low forward signal power, the non-reciprocity could be effectively activated due to the resonance-enhanced intracavity power.

3. The isolation could be sustained by a low forward power since the backward laser power is significantly suppressed by the high-reflectivity mirror at port 2.

For instance, for moderate non-reciprocal light absorption in the cell as $\mathcal{I} = 20\,\mathrm{dB}$, and assuming the intrinsic loss $\kappa_0 = 0$, we could estimate $T_{\mathrm{cav},+} = 0.176$, $P_{\mathrm{cav},+} = 44.2P_{\mathrm{in,f}}$ for the forward signal, and $T_{\mathrm{cav},-} = 3.3\times 10^{-6}$, $P_{\mathrm{cav},-} = 0.004P_{\mathrm{in,b}}$ for the backward probe. Therefore, the intracavity power for forward and backward inputs are different by approximately 10^4 times, which explains the isolation results presented in the main text Fig. 4D. However, due to the intrinsic losses of the practical cavity setup and the imperfect mode matching between the free-space input laser beam and the cavity mode, the practical enhancement factor is lower.

II. THEORETICAL DERIVATIONS

In this section, the theoretical derivation of the self-induced non-reciprocity and the nonlinear response of the non-reciprocal medium are provided. Both the microscopic theory of the light-atom interactions by the master equations and the macroscopic description of the non-reciprocal optical medium are studied. The corresponding non-reciprocal light propagation in the NLNR medium and the isolation are analytically derived and numerically investigated, which agrees with our experimental results presented in the main text.

A. The microscopic theory

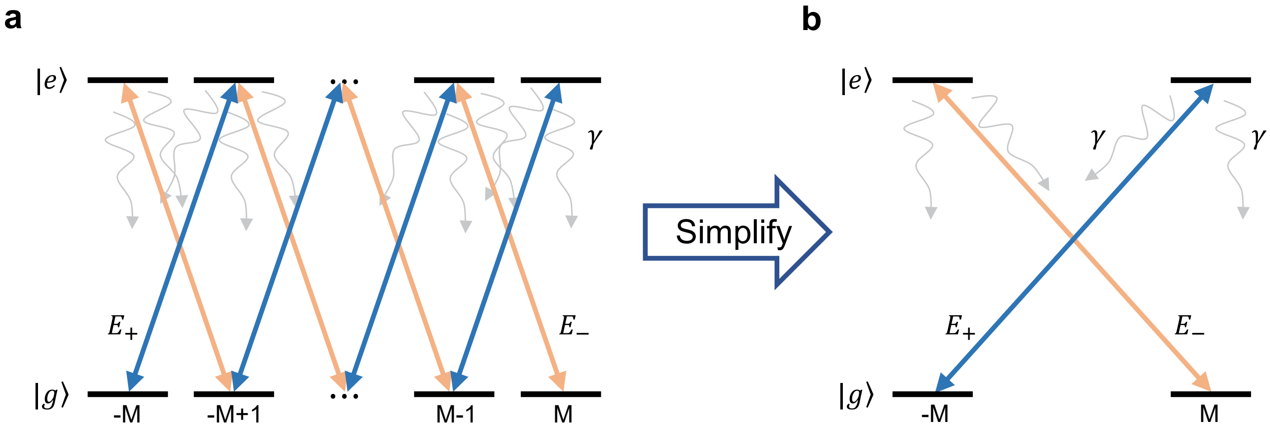

Figure S5. **a**, Schematic diagram of transition energy levels under the driving of both the σ^+-polarized light E_+ and the σ^--polarized light E_-. **b**, The simplified four-level model.

In our experiment, the atom vapor provides the medium for realizing nonlinear non-reciprocity, which originates from the nonlinear responses of the atoms. Treating the atoms as an optical medium, the polarization of the medium due to the input optical field could be obtained by solving quantum mechanical equation [2]. From the view at the microscopic level, the susceptibility of the medium is mainly determined by the atomic or molecular transitions. As shown in Fig. S5a, we consider a general atomic energy level diagram with a target optical transition between ground states ($|g\rangle$) and excited states ($|f\rangle$) that are near-resonant with the input light, and other energy levels of the atom are omitted since their transition frequency is far-detuned. As explained in the main text, circular dichroism and birefringence are the kernel optical properties for realizing non-reciprocity, thus the response difference of the atom with respect to the σ^+- and σ^--polarized light is essential. For the energy level presented in Fig. S5a, such a response difference could be achieved with a nonuniform population distribution on the ground Zeeman levels $-M, -M+1, ..., M-1, M$ and/or nonuniform transition strength between the ground and excited Zeeman levels. The selection rule allows the transition by decomposing the input light field into circularly polarized field intensities E_+ and E_-. Without losing generality of the theoretical model, we further simplify the model to two degenerate Zeeman levels $-M, M$, and the corresponding equivalent model is simplified in Fig. S5b, by which the essential physics of circular dichroism and birefringence could still be realized when the atom is polarized to the ground M or $-M$ energy level. We should stress that although the realistic atom energy level diagrams are rich, as the excited states might have spin numbers larger or lower than that of the ground state, our simplified model captures the main physics of our self-induced optical non-reciprocity mechanism.

The corresponding Hamiltonian for the simplified model can be written as ($\hbar = 1$)

$$H = \sum_{m=\mathrm{g,f}} \omega_m \left(\sigma_{mm}^{-M,-M} + \sigma_{mm}^{M,M}\right) + \left[\left(\Omega_+ \sigma_{\mathrm{gf}}^{-M,M} + \Omega_- \sigma_{\mathrm{gf}}^{M,-M}\right) e^{i\omega_d t}/2 + \mathrm{H.c.}\right], \tag{S.16}$$

where $\Omega_\pm = \mu_{\mathrm{gf}} E_\pm/\hbar$ is the Rabi frequency with the electric dipole moment μ_{gf}, $\sigma^{\mathrm{gf}} = |g\rangle\langle f|$ is the transition

operator, and ω_d is the driving frequency of the polarized field. Here, we have assumed that it is the degenerate level for the atom. Considering the decoherence of the atom, the dynamics of the system are governed by the Master equation [3], which reads

$$\frac{d\rho}{dt} = -i\left[H,\rho\right] + \gamma\left\{\mathcal{L}\left(\sigma_{\text{gf}}^{M,-M}\right) + \mathcal{L}\left(\sigma_{\text{gf}}^{-M,-M}\right) + \mathcal{L}\left(\sigma_{\text{gf}}^{M,M}\right) + \mathcal{L}\left(\sigma_{\text{gf}}^{-M,M}\right)\right\}. \tag{S.17}$$

Here, the Lindblad super-operator is defined as

$$\mathcal{L}\left(o\right) = o\rho o^{\dagger} - \rho o^{\dagger} o/2 - o^{\dagger} o \rho/2. \tag{S.18}$$

Here the total effective population decay rate is $\gamma = \gamma_0 + \gamma_{col}$ with both the natural decay rate γ_0 and the collision-induced relaxation rate γ_{col}. Note that we have assumed the same decay rate for both polarizations.

According to the Master equation, the dynamics of the system can be described by the following equations:

$$\frac{d}{dt}\sigma_{\text{ff}}^{M,M} = -i\frac{\Omega_+}{2}\left(\sigma_{\text{fg}}^{M,-M} - \sigma_{\text{gf}}^{-M,M}\right) - 2\gamma\sigma_{\text{ff}}^{MM}, \tag{S.19}$$

$$\frac{d}{dt}\sigma_{\text{ff}}^{-M,-M} = -i\frac{\Omega_-}{2}\left(\sigma_{\text{fg}}^{-M,M} - \sigma_{\text{gf}}^{M,-M}\right) - 2\gamma\sigma_{\text{ff}}^{-M,-M}, \tag{S.20}$$

$$\frac{d}{dt}\sigma_{\text{gg}}^{-M,-M} = -i\frac{\Omega_+}{2}\left(\sigma_{\text{gf}}^{-M,M} - \sigma_{\text{fg}}^{M,-M}\right) + \gamma\left(\sigma_{\text{ff}}^{M,M} + \sigma_{\text{ff}}^{-M,-M}\right), \tag{S.21}$$

$$\frac{d}{dt}\sigma_{\text{gg}}^{M,M} = -i\frac{\Omega_-}{2}\left(\sigma_{\text{gf}}^{M,-M} - \sigma_{\text{fg}}^{-M,M}\right) + \gamma\left(\sigma_{\text{ff}}^{M,M} + \sigma_{\text{ff}}^{-M,-M}\right), \tag{S.22}$$

$$\frac{d}{dt}\sigma_{\text{fg}}^{M,-M} = -i\Delta\sigma_{\text{fg}}^{M,-M} - i\frac{\Omega_+}{2}\left(\sigma_{\text{ff}}^{M,M} - \sigma_{\text{gg}}^{-M,-M}\right) - \gamma\sigma_{\text{fg}}^{M,-M}, \tag{S.23}$$

$$\frac{d}{dt}\sigma_{\text{fg}}^{-M,M} = -i\Delta\sigma_{\text{fg}}^{-M,M} - i\frac{\Omega_-}{2}\left(\sigma_{\text{ff}}^{-M,-M} - \sigma_{\text{gg}}^{M,M}\right) - \gamma\sigma_{\text{fg}}^{-M,M}, \tag{S.24}$$

$$\frac{d}{dt}\sigma_{\text{gg}}^{M,-M} = -i\frac{\Omega_+}{2}\sigma_{\text{gf}}^{M,M} + i\frac{\Omega_-}{2}\sigma_{\text{fg}}^{-M,-M}, \tag{S.25}$$

$$\frac{d}{dt}\sigma_{\text{fg}}^{M,M} = -i\Delta\sigma_{\text{fg}}^{M,M} + i\frac{\Omega_+}{2}\sigma_{\text{gg}}^{-M,M} - i\frac{\Omega_-}{2}\sigma_{\text{ff}}^{M,-M} - \gamma\sigma_{\text{fg}}^{M,M}, \tag{S.26}$$

$$\frac{d}{dt}\sigma_{\text{fg}}^{-M,-M} = -i\Delta\sigma_{\text{fg}}^{-M,-M} - i\frac{\Omega_+}{2}\sigma_{\text{ff}}^{-M,M} + i\frac{\Omega_-}{2}\sigma_{\text{gg}}^{M,-M} - \gamma\sigma_{\text{fg}}^{-M,-M}, \tag{S.27}$$

$$\frac{d}{dt}\sigma_{\text{ff}}^{-M,M} = -i\frac{\Omega_+}{2}\sigma_{\text{fg}}^{-M,-M} + i\frac{\Omega_+}{2}\sigma_{\text{gf}}^{M,M}. \tag{S.28}$$

Here, $\Delta = \omega_g - \omega_f + \omega_d$ is the laser detuning with respect to the transition. For the ensemble of atoms that interact with the light at a time-scale much longer than the relaxation time of the excited states, the response of the medium could be effectively approximated by the steady-state solutions of the system. The corresponding transition amplitudes of the atom are

$$\sigma_{\text{fg}}^{M,-M} = \frac{i\Omega_+\Omega_-^2\left(\gamma - i\Delta\right)/2}{\Omega_+^2\Omega_-^2 + \left(\gamma^2 + \Delta^2\right)\left(\Omega_+^2 + \Omega_-^2\right)}, \tag{S.29}$$

$$\sigma_{\text{fg}}^{-M,M} = \frac{i\Omega_-\Omega_+^2\left(\gamma - i\Delta\right)/2}{\Omega_+^2\Omega_-^2 + \left(\gamma^2 + \Delta^2\right)\left(\Omega_+^2 + \Omega_-^2\right)}, \tag{S.30}$$

which determines the polarization of the medium. According to the corresponding macroscopic description in the following section, the refractive index, dispersion and absorption of the field can be obtained.

B. The macroscopic description

In general, the polarization of materials driven by an external input field $\mathbf{E}$ can be written as $\mathbf{P} = \epsilon_0 \overleftrightarrow{\chi} \cdot \mathbf{E}$ [4, 5], where ϵ_0 is the vacuum permittivity and $\overleftrightarrow{\chi}$ is the susceptibility tensor. For isotropic media, the susceptibility tensor can be reduced to a scalar number, so the polarization vector is proportional to the input field vector $\mathbf{E}$. For the circular dichroism and birefringence material studied in this work, its susceptibility can be reduced to a diagonal

matrix as

$$\begin{pmatrix} P_+ \\ P_- \end{pmatrix} = \epsilon_0 \begin{pmatrix} \chi_+ & 0 \\ 0 & \chi_- \end{pmatrix} \begin{pmatrix} E_+ \\ E_- \end{pmatrix} \tag{S.31}$$

for the $\sigma^{\pm}$ polarization traveling field along the z axis. Here, the polarization $\mathbf{P}$ is the electric dipole moment per unit volume, and the driving field $\mathbf{E}$ is the macroscopic electric field intensity, which are both macroscopic quantities. Such macroscopic description of the medium by the susceptibility $\chi_\pm$ could be derived from the microscopic theory as [3]

$$\chi_\pm = \frac{\rho\,|\mu_{\rm gf}|}{\varepsilon_0}\frac{2\sigma_{\rm fg}^{\pm M,\mp M}}{E_\pm}. \tag{S.32}$$

Here, ρ is the atomic density, and $\mu_{\rm gf}$ is the electric dipole moment.

For circular dichroism and birefringence, we have $\chi_+ \neq \chi_-$ when $E_+ \neq E_-$, which means that we can realize different dispersion or absorption for different circularly polarized fields. The representation of the susceptibility can be transformed to Cartesian coordinates as follows:

$$E_\pm = \frac{1}{\sqrt{2}}\left(E_x \pm iE_y\right), \tag{S.33}$$

$$P_\pm = \frac{1}{\sqrt{2}}\left(P_x \pm iP_y\right), \tag{S.34}$$

and therefore we have

$$\begin{aligned} \begin{pmatrix} P_x \\ P_y \end{pmatrix} &= \epsilon_0 \frac{1}{\sqrt{2}} \begin{pmatrix} 1 & 1 \\ -i & i \end{pmatrix} \begin{pmatrix} \chi_+ & 0 \\ 0 & \chi_- \end{pmatrix} \frac{1}{\sqrt{2}} \begin{pmatrix} 1 & i \\ 1 & -i \end{pmatrix} \begin{pmatrix} E_x \\ Ey \end{pmatrix} \\ &= \epsilon_0 \begin{pmatrix} \frac{\chi_+ + \chi_-}{2} & \frac{i(\chi_+ - \chi_-)}{2} \\ \frac{-i(\chi_+ - \chi_-)}{2} & \frac{\chi_+ + \chi_-}{2} \end{pmatrix} \begin{pmatrix} E_x \\ Ey \end{pmatrix} \\ &= \epsilon_0 \begin{pmatrix} \chi_{xx} & i\chi_{xy} \\ -i\chi_{xy} & \chi_{yy} \end{pmatrix} \begin{pmatrix} E_x \\ Ey \end{pmatrix}. \end{aligned} \tag{S.35}$$

Here, $\chi_{xx} = \chi_{yy}$, and the off-diagonal susceptibility can be written as

$$\begin{aligned} \chi_{xy} &= \frac{\chi_+ - \chi_-}{2} \\ &= \frac{\rho\,|\mu_{\rm gf}|^2}{\varepsilon_0 \hbar} \frac{i\left(\Omega_-^2 - \Omega_+^2\right)(\gamma - i\Delta)/2}{\Omega_+^2\Omega_-^2 + (\gamma^2+\Delta^2)\left(\Omega_+^2 + \Omega_-^2\right)}. \end{aligned} \tag{S.36}$$

It is obvious that the difference in the susceptibility for circularly polarized light is proportional to $E_-^2 - E_+^2 = -2iE_xE_y$. In fact, the chirality of light is usually described by the spin angular momentum density [6, 7]

$$\mathbf{S} \propto \sigma\frac{\mathbf{k}}{|\mathbf{k}|} \propto \mathrm{Im}\left(\mathbf{E}^* \times \mathbf{E} + \mathbf{H}^* \times \mathbf{H}\right), \tag{S.37}$$

where σ is the chirality of light and $\mathbf{k}$ is the wave vector. Here, consider that the electric part along the z axis is $(\mathbf{E}^* \times \mathbf{E}) \cdot \mathbf{e}_z = 2iE_xE_y = \left(E_+^2 - E_-^2\right)$. It is obvious that the difference in the susceptibility is proportional to the spin angular momentum density $\mathbf{S}$, which is also related to the direction of transmission of the propagating circular-polarized light.

In addition to the linear polarization, the entire polarization generally includes nonlinear polarization, which means that the susceptibility needs to be written as a function of input fields $\overleftrightarrow{\chi} = \overleftrightarrow{\chi}(\mathbf{E})$. By including the non-reciprocal response from conventional magneto-optical rotation, the off-diagonal susceptibility can be expanded as

$$\chi_{xy} = \chi_{xy}^{(1)}\mathbf{B}\cdot\mathbf{e}_z + \chi_{xy}^{(3)}(\mathbf{E}\times\mathbf{E}^*)\cdot\mathbf{e}_z + ..., \tag{S.38}$$

where ... denotes the higher order expansion about E in the denominator of χ_{xy}. From our experimental results with

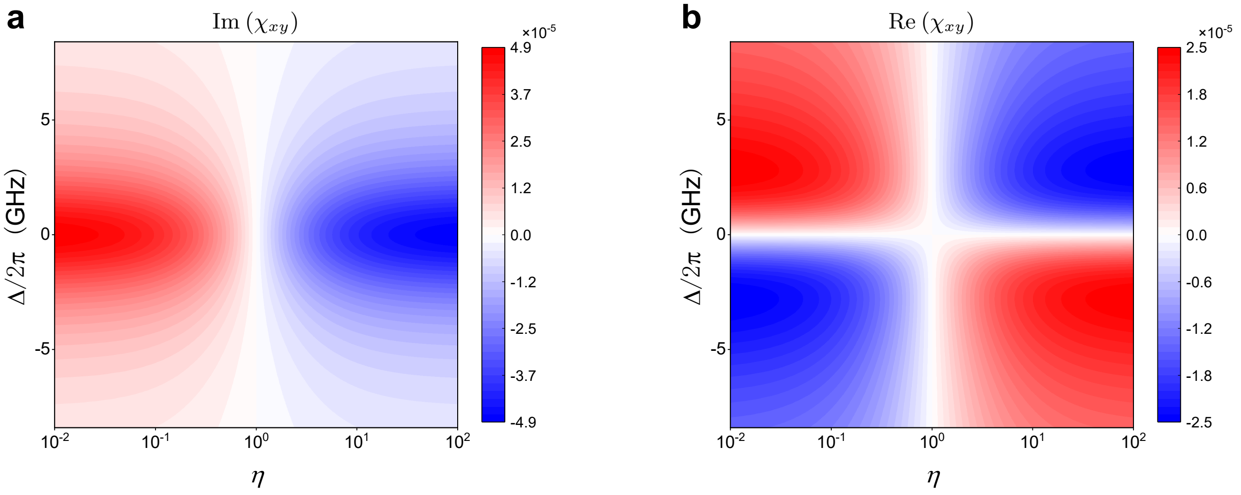

Figure S6. The imaginary [Im (χ_{xy})] (**a**) and real [Re (χ_{xy})] (**b**) parts of the off-diagonal susceptibility for an ensemble of atoms, with the energy diagram shown in Fig. S5b. Here, the ratio $\eta = \Omega_+^2/\Omega_-^2$ denotes the polarization of the input power, and Δ is the detuning between the signal and the transition energy. The related parameters are $\Omega_-/2\pi = 30$ MHz, $\gamma/2\pi = 2.8$ GHz, $\rho = 8.4 \times 10^{18}/\mathrm{m}^3$, $\mu_{gf} = 1.4 \times 10^{-29}$ C $\cdot$ m, $\hbar = 1.055 \times 10^{-34}$ J $\cdot$ s, and $\varepsilon_0 = 8.85 \times 10^{-12}$ F/m.

a light intensity of $I \approx 10^5 \mathrm{W/m}^2$, the Kerr-like nonlinear refractive index for the non-reciprocity coefficient is [2]

$$n_2 = \frac{1}{4n_0 \mathrm{Re}\,(n_0)\,\epsilon_0 c}\chi_{xy}^{(3)} = \frac{1}{4n_0 \mathrm{Re}\,(n_0)\,\epsilon_0 c}\frac{\chi_{xy}\epsilon_0 c}{2I} \approx 6 \times 10^{-11}\,\mathrm{m}^2/\mathrm{W}, \tag{S.39}$$

which is approximately nine orders of magnitude stronger than the conventional Kerr nonlinear index of dielectric materials (such as $2.19 \times 10^{-20}\,\mathrm{m}^2/\mathrm{W}$ for silica [8]).

In our experiment, we investigated the non-reciprocal transmission of light without an external magnetic field, i.e., $B = 0$ and demonstrated self-induced nonlinear non-reciprocal susceptibility ($\chi_{xy}^{(3)}$). As discussed in the main text, the real and imaginary parts of $\chi_{xy}^{(3)}$ could contribute to the circular dichroism and birefringence when there is a circularly polarized input light. As shown by Eq. (S.36), the susceptibility is not only a nonlinear function determined by the input field but also depends on the detuning Δ. To discuss the nonlinear function for the polarized field $\Omega_\pm$ and the detuning Δ, we plot the susceptibility as a function of the circular-polarized field. In Fig. S6, the susceptibility as a function of the ratio of the input circular polarized optical field component $\eta = \Omega_+^2/\Omega_-^2$ and the detuning Δ is presented. We noticed that the imaginary and real parts of the susceptibility are anti-symmetric about the ratio η, and the absorption reaches the maximum when the signal is on resonance with the atoms $\Delta = 0$. These results verify that the non-reciprocity ($|\chi_{xy}|$) is induced by polarized input when $\eta \neq 1$ and is enhanced when near-resonant with the atom transitions.

C. Non-reciprocal light propagation

Based on the self-induced non-reciprocal mechanism, we can realize the non-reciprocal propagation of the circular-polarized signal by the atomic medium. Considering the slowly varying envelop approximation, the dynamic evolution of the optical field can be given by the equation [3]

$$\frac{1}{c}\frac{\partial \mathbf{E}}{\partial t} + \frac{\partial \mathbf{E}}{\partial z} = ik\frac{\mathbf{P}}{\epsilon_0}, \tag{S.40}$$

where c is the vacuum speed of light and $k = |\mathbf{k}|$ is the wave vector of the signal field. Considering the steady state of the system, the propagation of the signal along the z-axis can be rewritten as

$$\frac{\partial E_\pm}{\partial z} = ik\chi_\pm E_\pm. \tag{S.41}$$

If $\chi_{+(-)}$ is a constant, we obtain $E_{\pm}(z) = E_{\pm}(0)\, e^{ik\chi_{\pm} z}$. For the same input signal, the non-reciprocal propagation is $E_{+}(z)/E_{-}(z) = e^{2ik\chi_{xy} z}$, and the corresponding non-reciprocal transmission and phase are $|E_{+}(z)/E_{-}(z)|^{2} = e^{-4k\mathrm{Im}(\chi_{xy})z}$ and $\phi = 2k\mathrm{Re}(\chi_{xy})\, z$, respectively.

In practical experiments, the susceptibility $\chi_{\pm}$ is a complex function of the field $\mathbf{E}$. Considering the thermal distribution of the atom velocity (v) for the atomic vapor, the Doppler effect due to the motion $\Delta \to \Delta + kv$ should be included in the calculation. Here, we focus on the absorption of the circular polarized signal, and the propagation equations become

$$\frac{\partial E_{+}}{\partial z} = -k\zeta \int_{-\infty}^{+\infty} p(v) \frac{\Omega_{-}^{2}\gamma}{\Omega_{+}^{2}\Omega_{-}^{2} + \left(\gamma^{2} + (\Delta + kv)^{2}\right)\left(\Omega_{+}^{2} + \Omega_{-}^{2}\right)} E_{+} dv, \tag{S.42}$$

$$\frac{\partial E_{-}}{\partial z} = -k\zeta \int_{-\infty}^{+\infty} p(v) \frac{\Omega_{+}^{2}\gamma}{\Omega_{+}^{2}\Omega_{-}^{2} + \left(\gamma^{2} + (\Delta + kv)^{2}\right)\left(\Omega_{+}^{2} + \Omega_{-}^{2}\right)} E_{-} dv, \tag{S.43}$$

where $\zeta = |\mu_{gf}|^{2}/\varepsilon_{0}/\hbar$ and $p(v) = \rho e^{-v^{2}/v_{p}^{2}}/(v_{p}\sqrt{\pi})$ are the Maxwell Boltzmann velocity distributions, $v_{p} = \sqrt{2k_{B}T/m}$ is the most likely velocity, k_{B} is the Boltzmann constant, T is the absolute temperature of the vapor cell and m is the atomic mass. For convenience, we derive the evolution of the corresponding Rabi frequencies $\Omega_{\pm}$, and the propagation equations can be written as

$$\frac{\partial \Omega_{+}}{\partial z} = -k\zeta \int_{-\infty}^{+\infty} p(v) \frac{\Omega_{-}^{2}\gamma}{\Omega_{+}^{2}\Omega_{-}^{2} + \left(\gamma^{2} + (\Delta + kv)^{2}\right)\left(\Omega_{+}^{2} + \Omega_{-}^{2}\right)} \Omega_{+} dv, \tag{S.44}$$

$$\frac{\partial \Omega_{-}}{\partial z} = -k\zeta \int_{-\infty}^{+\infty} p(v) \frac{\Omega_{+}^{2}\gamma}{\Omega_{+}^{2}\Omega_{-}^{2} + \left(\gamma^{2} + (\Delta + kv)^{2}\right)\left(\Omega_{+}^{2} + \Omega_{-}^{2}\right)} \Omega_{-} dv, \tag{S.45}$$

The ratio of the absorption parameters for the signals is $\mathrm{Im}(\chi_{+})/\mathrm{Im}(\chi_{-}) = \Omega_{-}^{2}/\Omega_{+}^{2}$, which means that the non-reciprocity depends on the ratio of the input circularly polarized optical field components $\eta = \Omega_{+}^{2}/\Omega_{-}^{2}$. In our experiment, we have $\Omega_{\pm} \ll \gamma$, especially for the vapor cell filled with buffer gas, in which $\gamma \approx 2\pi \times 2.8$ GHz due to atomic collision. To realize self-induced non-reciprocal propagation, the input field should be circularly polarized $\Omega_{+} \ll \Omega_{-}$, and we obtain

$$\Omega_{+}(z) = \Omega_{+}(0) \exp\left[-k\zeta z \int_{-\infty}^{+\infty} p(v) \frac{\gamma}{\gamma^{2} + (\Delta + kv)^{2}} dv\right], \tag{S.46}$$

which shows that the transmission

$$T_{+} = \left|\frac{E_{+}(z)}{E_{+}(0)}\right|^{2} = \left|\frac{\Omega_{+}(z)}{\Omega_{+}(0)}\right|^{2} \tag{S.47}$$

decreases exponentially with the transmission distance z and $T_{+} \approx \exp(-2k\zeta z\rho/\gamma)$ when $\Delta = 0$ and $kv_{p} \ll \gamma$. However, the corresponding transmission for the circularly polarized signal E_{-} is $T_{-} \approx \exp(-2k\zeta z\rho\eta/\gamma)$, and therefore, we have $T_{-} \approx 1$ for the transmission distance $z \ll \gamma/(2k\zeta\rho\eta)$.

Furthermore, we have to consider the population relaxation effect of the ground Zeeman states, which is due to the thermal motion of atoms that move into or out of the area of the driving field in our experiment. By including the incoherent ground state relaxation process, the Master equation can be rewritten as

$$\frac{d\rho'}{dt} = \frac{d\rho}{dt} + J\left\{\mathcal{L}\left(\sigma_{\mathrm{gg}}^{M,-M}\right) + \mathcal{L}\left(\sigma_{\mathrm{gg}}^{-M,M}\right)\right\}, \tag{S.48}$$

where J is the relaxation coefficient of the ground states. By solving the steady-state solution, we obtain

$$\sigma_{\mathrm{fg}}^{M,-M} = \frac{i\Omega_{+}\left(\gamma - i\Delta\right)\left[\Omega_{-}^{2} + \frac{4J\left(\gamma^{2}+\Delta^{2}+\Omega_{-}^{2}/4\right)}{\gamma}\right]/2}{\Omega_{+}^{2}\Omega_{-}^{2} + \left(\gamma^{2}+\Delta^{2}\right)\left(\Omega_{+}^{2}+\Omega_{-}^{2}\right) + \frac{4J\left[\left(\gamma^{2}+\Delta^{2}\right)\left(2\gamma^{2}+2\Delta^{2}+3\Omega_{+}^{2}/4+3\Omega_{-}^{2}/4\right)+\Omega_{+}^{2}\Omega_{-}^{2}/4\right]}{\gamma}}, \tag{S.49}$$

$$\sigma_{\mathrm{fg}}^{-M,M} = \frac{i\Omega_{-}\left(\gamma - i\Delta\right)\left[\Omega_{+}^{2} + \frac{4J\left(\gamma^{2}+\Delta^{2}+\Omega_{+}^{2}/4\right)}{\gamma}\right]/2}{\Omega_{+}^{2}\Omega_{-}^{2} + \left(\gamma^{2}+\Delta^{2}\right)\left(\Omega_{+}^{2}+\Omega_{-}^{2}\right) + \frac{4J\left[\left(\gamma^{2}+\Delta^{2}\right)\left(2\gamma^{2}+2\Delta^{2}+3\Omega_{+}^{2}/4+3\Omega_{-}^{2}/4\right)+\Omega_{+}^{2}\Omega_{-}^{2}/4\right]}{\gamma}}. \tag{S.50}$$

Then we can further obtain the corresponding susceptibility $\chi_{\pm}$ with the relaxation coefficient J, and the signal transmission and non-reciprocity can be numerically calculated by the propagation equations.

D. Cavity-enhanced non-reciprocity

In previous discussions, we noticed that the transmission is greatly influenced by the ratio $\eta = \Omega_{+}^{2}/\Omega_{-}^{2}$. In particular, when the value of the ratio is extremely large or extremely small, that is, $\eta \ll 1$ or $\eta \gg 1$, then we can observe very strong non-reciprocity. Naturally, an asymmetric bow-tie cavity is introduced into our experimental system, and we place the atomic cell into the asymmetric cavity, as shown in Fig. S7. The forward and backward ports of the cavity are labeled by M1 and M2, and the corresponding reflectivities are $R_1 = 91.8\%$ and $R_2 = 99.6\%$, respectively. The non-reciprocal optical leverage is rooted in the difference in the injected energy $\left(1 - R_{1,2}\right) P_{\mathrm{in,f(b)}}$ from the forward and backward ports of the traveling wave cavity, and this absorption difference is further amplified when the signal light travels through the atomic vapor due to the different susceptibilities for different polarized lights, which depend on the ratio η. The corresponding propagation equation still satisfies $\frac{\partial E_{\mathrm{f}}}{\partial z} = ik\chi_{\mathrm{f}}E_{\mathrm{f}}$ when the forward signal passes through the atomic vapor with length $l = 1\,\mathrm{cm}$. Note that in this section and in the main text, we only use the letter f to denote the forward signal, which is σ^{+}-polarized according to our experimental setup (Extended Data Fig. 2a).

From Fig. S7, we also consider the intrinsic loss of the cavity (due to the absorption of the cell glass and imperfect surfaces), which could be effectively described by a propagation loss that can be rewritten as $E_{\mathrm{f}}\left(l\right) \rightarrow \sqrt{1 - R_0}E_{\mathrm{f}}\left(l\right)$, with $R_0 = 0.74$ for our experiments. The output signal is derived as $E_{\mathrm{out,f}} = \sqrt{\left(1 - R_2\right)\left(1 - R_0\right)}E_{\mathrm{f}}\left(l\right)$. Meanwhile, the reflected signal passes through the flat mirrors M_3 and M_4 in turn, and we obtain $\widetilde{E}_{\mathrm{f}}\left(l\right) = \sqrt{R_2\left(1 - R_0\right)}E_{\mathrm{f}}\left(l\right)$. For a traveling wave cavity, the condition $E_{\mathrm{f}}(0) = \sqrt{1 - R_1}E_{\mathrm{in,f}} + \sqrt{R_1}\widetilde{E}_{\mathrm{f}}\left(l\right)$ is satisfied when the system is at the steady state, and the corresponding transmission and input power can be written as

$$\left|\frac{E_{\mathrm{out,f}}}{E_{\mathrm{in,f}}}\right|^{2} = \left|\frac{\sqrt{\left(1 - R_1\right)\left(1 - R_2\right)\left(1 - R_0\right)}E_{\mathrm{f}}\left(l\right)}{E_{\mathrm{f}}\left(0\right) - \sqrt{R_1R_2\left(1 - R_0\right)}E_{\mathrm{f}}\left(l\right)}\right|^{2}, \tag{S.51}$$

$$P_{\mathrm{in,f}} \propto \left|\frac{E_{\mathrm{f}}\left(0\right) - \sqrt{R_1R_2\left(1 - R_0\right)}E_{\mathrm{f}}\left(l\right)}{\sqrt{1 - R_1}}\right|^{2}. \tag{S.52}$$

It is obvious that we can easily obtain the transmission and input power for the backward signal by exchanging R_1 and R_2 in the above expressions. In addition to the non-reciprocity induced by the difference from the susceptibility $\chi_{f(b)}$, we also find that an asymmetric cavity induces $P_{\mathrm{in,f}}/P_{\mathrm{in,b}} = (1-R_2)/(1-R_1) \approx 0.049$ for the same susceptibility of the forward and backward signals, that is, the transmission is the same value, which shows cavity-enhanced non-reciprocal leverage.

E. Design of the optical circulator based on the NLNR medium

There are two simple schemes to implement the circulators with the non-reciprocal device. One scheme is to employ the dispersion effect of the atomic esenmble. In our previous work [9], we had used a vapor cell without buffer gas to realize non-reciprocity by utilizing the dispersion effect. The resonance frequencies of σ^{+} and σ^{-} light for the cavity are different due to the dispersion of the polarized atomic ensemble, and our circulator scheme is based on this. Four ports on two PBSs are defined in Fig. S8. The atomic esenmble are polarized by another laser (not shown in figure), so the σ^{+} light will pass through the cavity while the σ^{-} light will be reflected. Light from port1 is converted into σ^{+} before entering the cavity, passes through, and reaches port2. Meanwhile, light from port2 is converted into σ^{-},

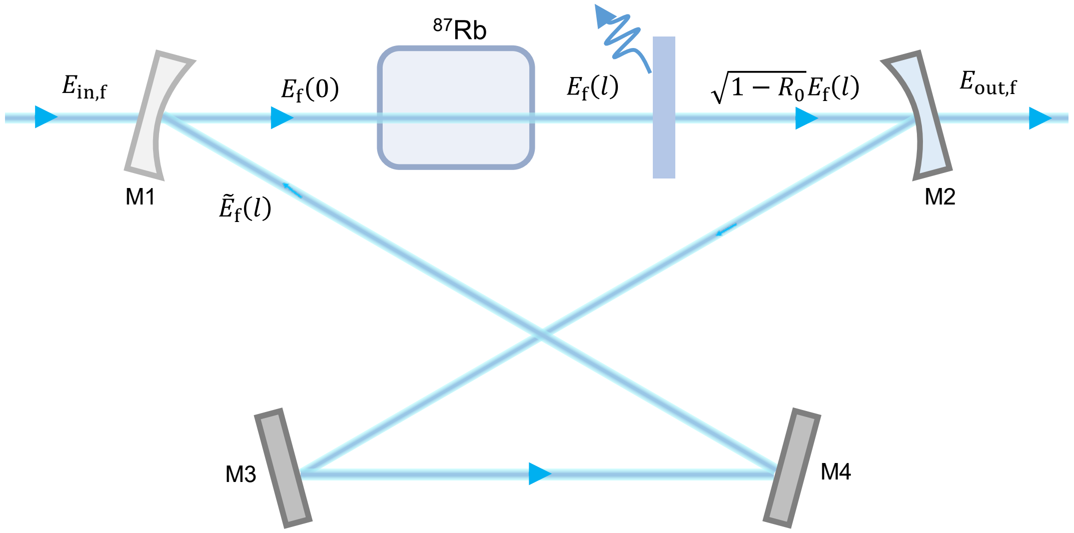

Figure S7. Schematic illustration of the traveling wave cavity for a forward signal, where M1 is the mirror with reflectivity $R_1 = 91.8\%$, M2 is the mirror with reflectivity $R_1 = 99.6\%$, M3 and M4 are plane mirrors, and the intrinsic loss of the cavity is $\kappa_0/2\pi = 10.8$ MHz, that is, $R_0 = 71.4\%$.

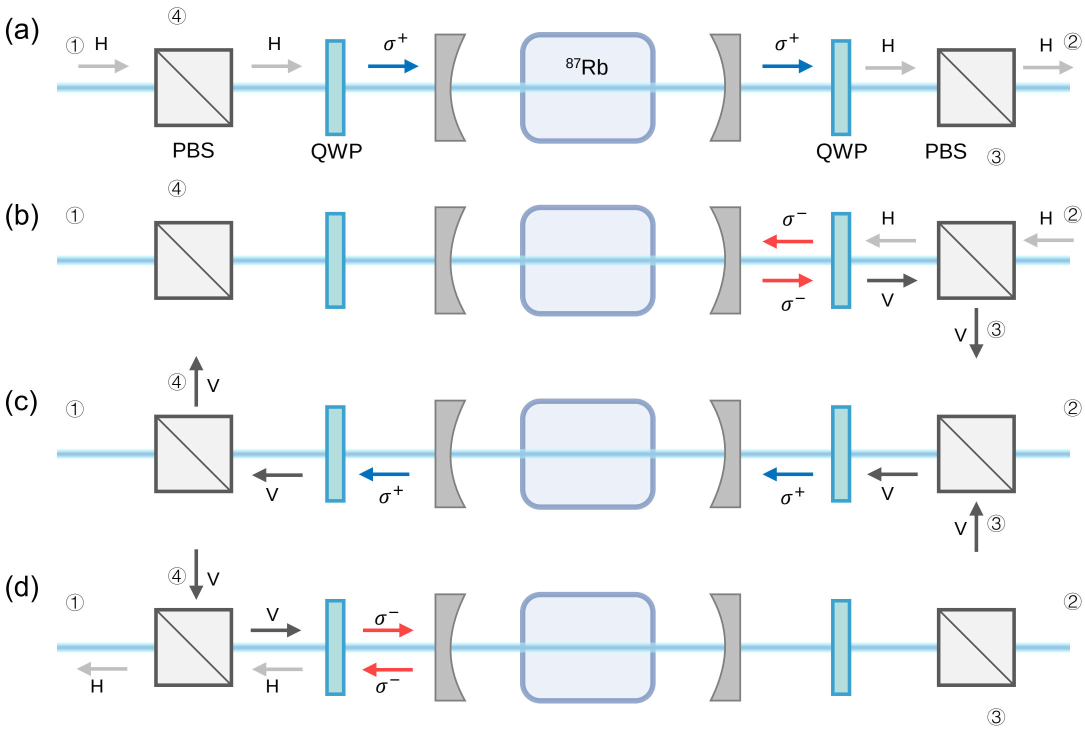

Figure S8. Schematic diagram of the circulator based on dispersion effect.

reflected by the cavity and reaches port3. Similarly, light from port3 will reach port4 and light from port4 will reach port1.

Another scheme is the absorption device, which are composed of three isolators, as shown in Fig. S9. This is a common circulator scheme for any kind of isolators. The isolator between port1 and port2 make sure that light from port1 can reach port2 while light from port2 cannot reach port1 due to the absorption effect, and the partial light from port2 can reach port3 but not the other way around. The defect of this scheme is that the maximum transmittance is limitted by the beam splitters.

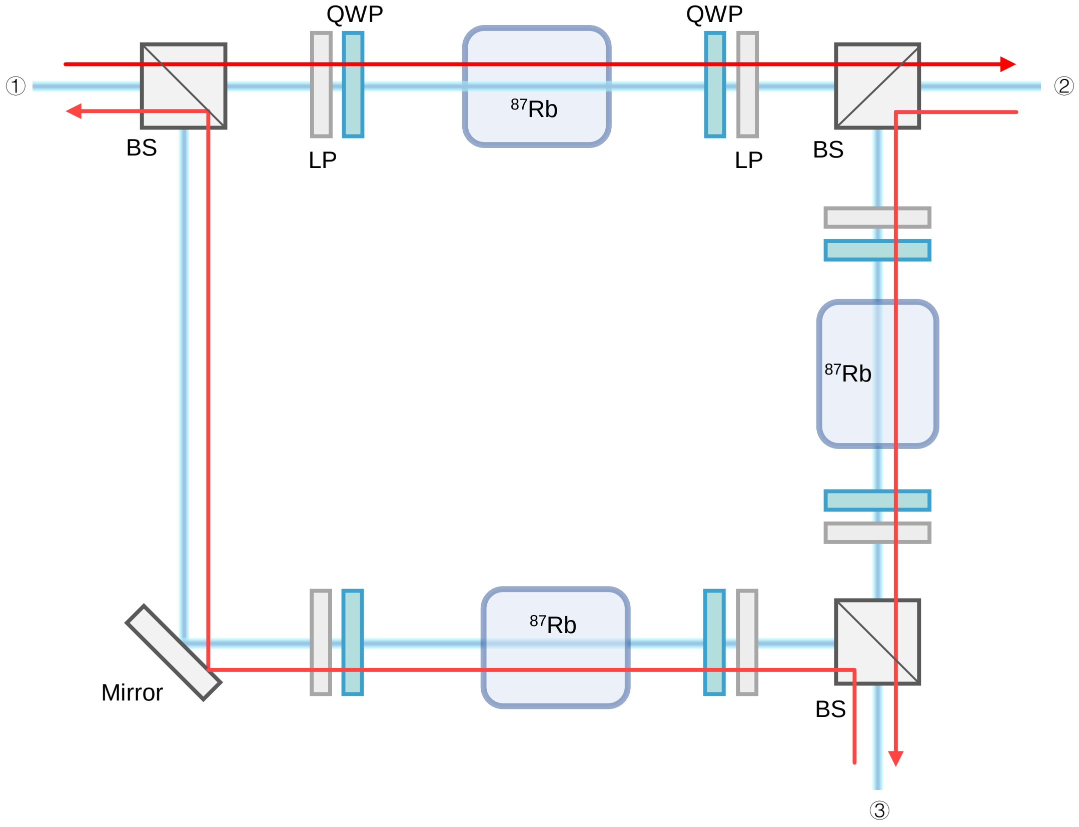

Figure S9. Schematic diagram of the circulator based on absorption effect.

F. Numerical Results

1. *Self-induced non-reciprocity without cavity*

The general property of the self-induced non-reciprocity is numerically verified. In Fig. S10a, we numerically calculate the transmission of the atomic medium T_+ as a function of the detuning Δ for the ratio $\eta = 0.1,\ 1,\ 2$ by the above propagation equations, with $J = 0$. The bandwidth of the transmission is fully determined by the decay rate γ due to the relatively small Doppler broadening (a few hundreds of MHz) compared to the collision-induced broadening effect ($\gamma/2\pi \sim 2.8\,\mathrm{GHz}$), even for the temperate $T \sim 100\,^\circ\mathrm{C}$ in our experiment. When $\eta \ll 1$, we observe that most of the circularly polarized signal E_+ is absorbed, which leads to $T_+ \approx 10^{-8}$ for the atomic vapor length $l = 1\,\mathrm{cm}$. With the increasing ratio when getting to $\eta = 1$, the absorption coefficient $\mathrm{Im}\left(\chi_+\right) \propto 1/\left(1+\eta\right)$ is reduced by half, which leads that the transmission increases by four orders of magnitude compared to $\eta \ll 1$. When $\eta = 2$, the σ^+-polarized light dominates, and we found that the absorption is negligible. From Eq. (S.53), we find that the propagation equation is a nonlinear function of the signal strengths. In Fig. S10b, the dependence of transmission on the ratio η is numerically investigated. The black line shows that the transmission has a sharp increase near $\eta \approx 1$ and remains almost unchanged when $\eta \ll 1$ or $\eta \gg 1$, which clearly reveals the nonlinear behavior. In Fig. S10b, we also study the influence of the relaxation effect on the non-reciprocal transmission. With the increase in J, the rapid changes around $\eta = 1$ are slowed down, as shown by the red and blue lines. Meanwhile, the minimum absorbance is also weakened for the parameters of our experimental setup. These numerical results are in good agreement with our experimental results. The isolation is calculated as $\mathcal{I} = \left|10\log_{10}\frac{T_+}{T_-}\right|$, which should be symmetric about the detuning Δ and η, as shown in Fig. S10c. These numerical results are consistent with our analysis.

In Fig. S11a, we numerically fit the experimental results in Fig. 3b of the main text based on our theoretical model above, where the signal passes through Cell2 and Cell1 in turn, and our propagation equations are also numerically calculated for two pieces of process . For more details about the experimental setup, see Sect. I D. The transmission $T_+^{(2)}$ for Cell2 as a function of the angle of the quarter plate θ is solved, and the corresponding parameters are $E_+ = E_0\cos\left(\theta+\frac{\pi}{4}-\delta\theta\right)$ and $E_- = E_0\sin\left(\theta+\frac{\pi}{4}-\delta\theta\right)$. with $\delta\theta$ being the deviation of the angle. Here, we introduce

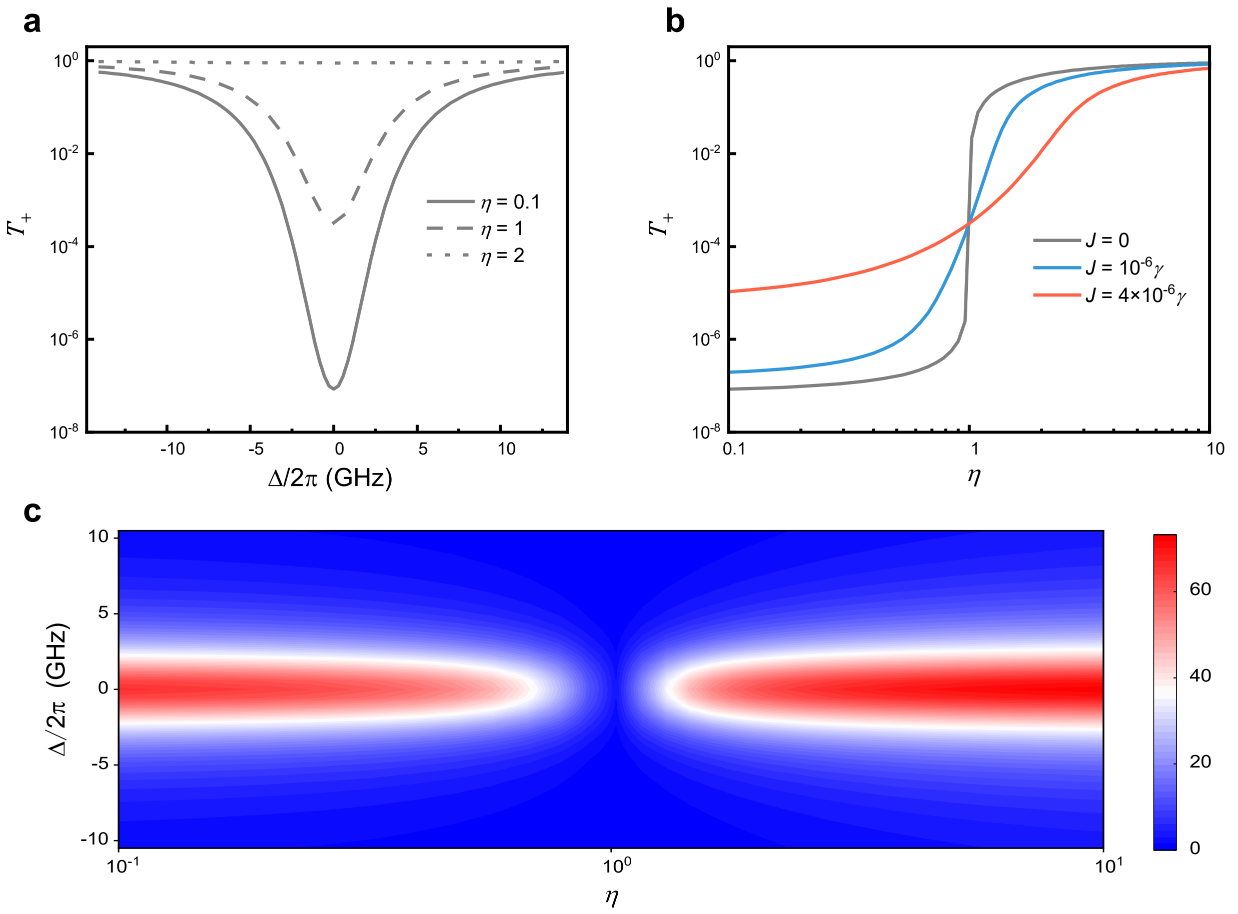

Figure S10. The transmission characteristic in th0e free space. The transmission as a function of the detuning Δ (**a**) without considering the relaxation effect of the atomic vapor ($J = 0$) and the ratio η (**b**) for the on-resonance condition $\Delta = 0$. **c**, Isolation versus detuning and the ratio with the relaxation coefficient $J = 10^{-6}\gamma$. The corresponding parameters are the wave vector $k = 2\pi/795\,\mathrm{nm}^{-1}$, the cell length $l = 1\,\mathrm{cm}$, and the temperature $T = 84\,^{\circ}\mathrm{C}$. The other parameters are the same as in Fig. S6.

a parameter $\delta\theta \approx 0.028\pi$ denoting the deviation of the angle to fit the experimental results, and such a deviation $\delta\theta$ may be due to the inaccuracy of the experimental manipulation and imperfect calibrations. The relaxation coefficient is $J = 6 \times 10^{-6}\gamma$. In Fig. S11b, we choose $\Omega_- = \Omega_+/100$ to fit the experimental results. The other parameters are the same as those in Fig. S10a.

According to the fitting parameters, our theory [Eq. (S.5)] predicts that ideal isolation reaches 70 dB when there is no background noise. When fitting the experimental results in Fig. 3b of the main text, the numerical results are presented by including background noise due to the lasers and detectors.

2. *Cavity-enhanced non-reciprocal leverage*

In this section, we numerically fit the experimental results for the cavity-enhanced non-reciprocal leverage when the forward laser is alone. The corresponding optical field propagation equation for $\Delta = \Omega_- = 0$ is

$$\frac{\partial E_\mathrm{f}}{\partial z} = -k\zeta \int_{-\infty}^{+\infty} p(v) \frac{4J}{\Omega_+^2 + \frac{4J\left[2\gamma^2 + 2(kv)^2 + 3\Omega_+^2/4\right]}{\gamma}} E_\mathrm{f} dv, \tag{S.53}$$

$$\approx -k\zeta \int_{-\infty}^{+\infty} p(v) \frac{4J}{\Omega_+^2 + 8J\gamma} E_\mathrm{f} dv. \tag{S.54}$$

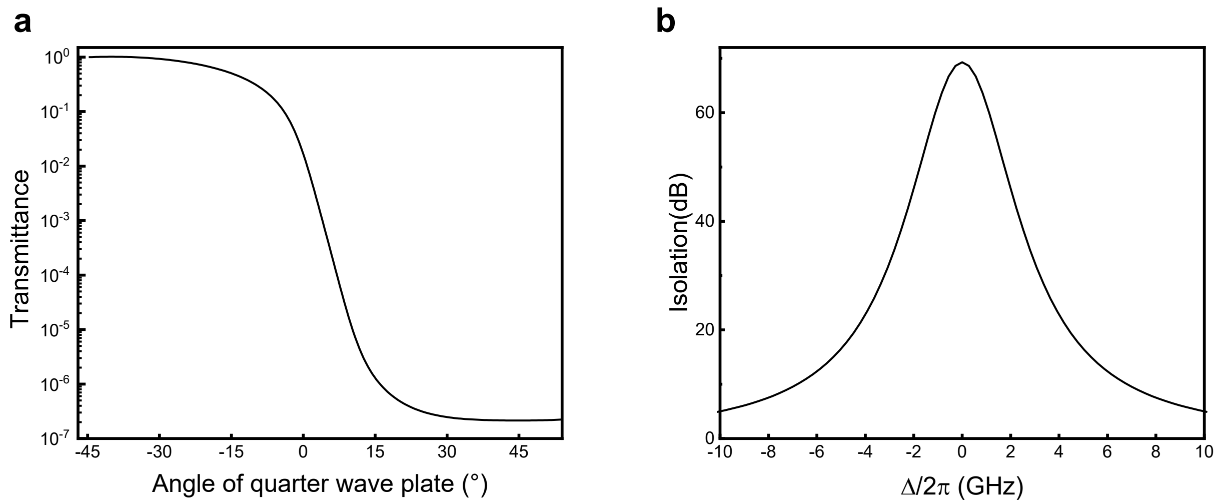

Figure S11. Numerical simulation of the experimental results in Fig. 3 in the main text based on propagation equations with the relaxation coefficient. All other parameters are taken from experiments.

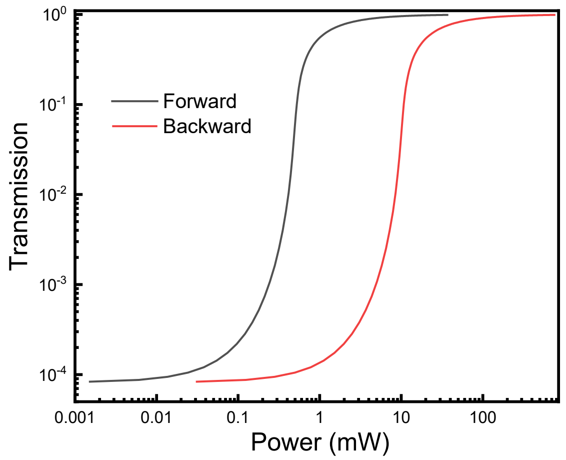

Figure S12. Numerical simulation of experimental results in Fig. 4 in the main text for the cavity-enhanced non-reciprocal leverage. The gray and red lines show the transmission when the forward or backward laser is alone.

In the last step, the approximation of $kv_p, \Omega+ \ll \gamma$ is adapted, which is a reasonable approximation of our experiments. It is obvious that we have $E_{\mathrm{f}}(l) \approx E_{\mathrm{f}}(0)\exp\left[-k\zeta\rho l/(2\gamma)\right]$ if $\Omega+^2 \ll 8J\gamma$, and the minimum transmission has nothing to do with the relaxation J. For the cavity case, we choose the relaxation parameter $J = 6 \times 10^{-5}\gamma$, which is larger than that for the case of free space since the laser beam waist in the cavity is smaller than that in the free space. The other parameters are the same as those in Fig. S6. The transmission for the backward signal alone is also investigated, and the results are shown by the red line in Fig. S12. Comparing the transmissions, the curve shape of the backward signal is the same as the curve of the forward signal, and only the location is shifted to the right as $P_{\mathrm{in,b}} = (1 - R_1)/(1 - R_2)P_{\mathrm{in,f}} \approx 20P_{in,f}$. Such behavior of the non-reciprocal leverage is consistent with our

experimental results [Fig. 4c in the main text].

[1] Romalis, M. V., Miron, E. & Cates, G. D. Pressure broadening of rb D_1 and D_2 lines by ^{3}he, ^{4}he, n_2, and xe: Line cores and near wings. *Phys Rev A* **56**, 4569–4578 (1997).

[2] Boyd, R. W. Nonlinear optics. Academic press (2020).

[3] Scully, M. O. & Zubairy, M. S. Quantum optics. American Association of Physics Teachers (1999).

[4] Momeni, A. et al. Generalized optical signal processing based on multioperator metasurfaces synthesized by susceptibility tensors. *Phys Rev Applied* **11**, 064042 (2019).

[5] Kauranen, M. et al. Quantitative determination of electric and magnetic second-order susceptibility tensors of chiral surfaces. *Phys Rev B* **55**, R1985–R1988 (1997).

[6] Bliokh, K. Y. & Nori, F. Transverse and longitudinal angular momenta of light. *Physics Reports* **592**, 1–38 (2015). Transverse and longitudinal angular momenta of light.

[7] Bliokh, K. Y. et al. Field theory spin and momentum in water waves. *Science Advances* **8**, eabm1295 (2022).

[8] Kabaciński, P. et al. Nonlinear refractive index measurement by spm-induced phase regression. *Opt Express* **27**, 11018–11028 (2019).

[9] Hu, X.-X. et al. Noiseless photonic non-reciprocity via optically-induced magnetization. *Nature Communications* **12**, 2389 (2021).
